# Screening of Medicines for Malaria Venture Open Boxes Identifies Potent SARS-CoV‑2 Papain-like Protease (PL^pro^) Inhibitors

**DOI:** 10.1021/acsomega.5c04858

**Published:** 2025-09-11

**Authors:** Victor Oliveira Gawriljuk, Gabriela Dias Noske, Rafaela Sachetto Fernandes, Aline Minalli Nakamura, Marjorie C. L. C. Freire, Mariana Ortiz Godoy, Vinicius Bonatto, Rafael Chelucci, Adriano Andricopulo, Malina A. Bakowski, Karen C. Wolff, Laura Riva, Jeremy N. Burrows, Timothy N. C. Wells, Benoît Laleu, Ronaldo Martins, Juliano Paula Souza, Eurico Arruda, Kirandeep Samby, Sujay Laskar, Rafael Victorio Carvalho Guido, Glaucius Oliva, Andre Schutzer Godoy

**Affiliations:** † São Carlos Institute of Physics, University of São Paulo, Av. João Dagnone, 1100—Jardim Santa Angelina, São Carlos 13563-120, Brazil; ‡ Calibr-Skaggs Institute for Innovative Medicine, La Jolla, California 92037, United States; § 127356 MMV Medicines for Malaria Venture, ICC, Route de Pré-Bois 20, Geneva 1215, Switzerland; ∥ Department of Cellular and Molecular Biology and Pathogenic Bioagents, Ribeirão Preto Medical School, University of São Paulo, Ribeirão Preto 14040-900, Brazil; ⊥ Departamento de Análises Clínicas, Toxicológicas e Bromatológicas, Faculdade de Ciências Farmacêuticas de Ribeirão Preto—FCFRP, 28133 Universidade de São Paulo—USP, Ribeirão Preto, São Paulo 14040-903, Brazil; # Johnson and Johnson Pvt., Ltd., Gurgaon 122003, India; ¶ TCG Lifesciences, West Bengal 700091, India

## Abstract

The COVID-19 pandemic, caused by SARS-CoV-2, has led to more than 760 million infections and 6.9 million deaths worldwide. While vaccines have played a crucial role in controlling the disease, emerging viral variants pose a threat to their long-term efficacy, highlighting the need for antiviral therapies. To accelerate drug discovery, Medicines for Malaria Venture (MMV) developed open-access compound libraries, including the malaria box, pathogen box, COVID box and, with the drugs for neglected disease initiative, pandemic response box, comprising nearly 1400 drug-like molecules. These resources have been widely used in phenotypic screens to identify potential SARS-CoV-2 inhibitors, but target-based screening remained unexplored, especially for targets such as papain-like protease (PL^pro^). Here, we report a target-based screening campaign against SARS-CoV-2 proteases, focusing on both the main protease (M^pro^) and PL^pro^. From this effort, MMV1634397 emerged as a promising PL^pro^ inhibitor (IC_50_ = 0.7 μM). Further optimization led to analogs with improved activity such as 5, 10, and 13, with IC_50_ values of 0.16, 0.34, and 0.06 μM, respectively. The most potent compound and its isomer also exhibited antiviral activity in ACE2-HeLa cells (EC_50_ = 2.9 and 3.7 μM, respectively) with favorable pharmacokinetic properties. Our findings highlight the value of the MMV open-access libraries in accelerating target-based antiviral drug discovery against SARS-CoV-2 and other emerging pathogens.

## Graphical abstract

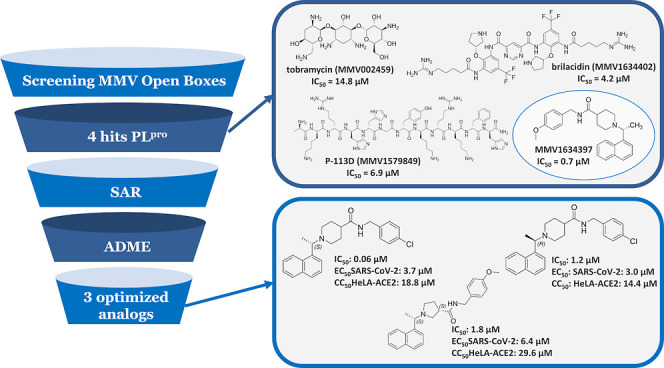

## 1 Introduction

The COVID-19 pandemic, caused by Severe Acute Respiratory Syndrome Coronavirus 2 (SARS-CoV-2), has resulted in over 760 million infection cases and 6.9 million deaths globally since its beginning at the end of 2019 World Health Organization (WHO). Most of the patients experience mild to moderate respiratory illness, however, severe cases include the development of pneumonia and acute respiratory distress syndrome, both associated with the relatively high mortality rate of this disease.^1^ Despite the WHO announcing the conclusion of its emergency phase in May 2023, the death of COVID-19 remains a global health burden.

While the implementation of vaccines stands as a pivotal public health measure in controlling disease, the emergence of SARS-CoV-2 variants poses a potential threat to the global success of mass vaccination campaigns. As a result, there is an urgent need to discover active compounds against SARS-CoV-2, including antiviral molecules that can be developed into effective treatments or preventive strategies against viral infections.

To assist the rapid development of new therapies for infectious diseases, Medicines for Malaria Venture (MMV) developed a series of open drug or drug-like compound libraries to catalyze new drug discovery in neglected diseases, including the malaria box (MB), the pathogen box (PB), the COVID box (CB), and, in partnership with the drugs for neglected disease initiative (DNDi), the pandemic response box (PRB), with almost 1400 molecules in total, which are part of MMV Open initiatives.^2–4^

Since the onset of the COVID-19 pandemic, several research groups have utilized MMV boxes to identify potential inhibitors of SARS-CoV-2 through phenotypic assays.^5–8^ However, target-based screening, which can lead to the identification of new potential inhibitors, has not been conducted to the same extent. Early in the pandemic, we initiated an enzyme-based high-throughput screening (HTS) of all Open Access Box compound series to discover inhibitors of SARS-CoV-2 proteases, including the main protease (M^pro^) and the papain-like protease (PL^pro^). M^pro^ and PL^pro^ are cysteine proteases that are crucial in viral replication. The former is a chymotrypsin-like capable of cleaving Pp1a/Pp1ab polyproteins at 11 sites (sequence consensus X-(L/F/M)-Q ↓ (G/A/S)-X),^9^ while the latter is a papain-like cysteine protease responsible for cleaving at 3 additional sites in the Pp1a/Pp1ab polyproteins (sequence consensus LXGG ↓ XX) to yield multiple functional proteins.^10^

The successful development of drugs targeting M^pro^ has overshadowed efforts to identify PL^pro^ inhibitors, resulting in a significant discovery gap between the two proteases.^11–14^ By inclusion of PL^pro^ in screening campaigns, this gap can begin to be addressed. In this context, the most potent initial hit, MMV1634397, inhibited PL^pro^ with an IC_50_ of 0.7 μM and showed measurable antiviral activity. Subsequent optimization led to analogs 12 and 13, which demonstrated improved potency with IC_50_ values of 1.2 and 0.06 μM, respectively. These compounds also exhibited antiviral activity in ACE2-HeLa-infected cells, with EC_50_ values of 2.9 and 3.7 μM, alongside encouraging preliminary pharmacokinetic profiles.

## 2 Materials and Methods

### 2.1 SARS-CoV-2 PL^pro^ Cloning, Expression, Purification, and Activity Assays

The pET28a plasmid encoding PL^pro^, as previously described,^15^ was used to transform *E. coli* BL21 cells. Cultures were grown in lysogen broth (LB) supplemented with 50 μg/mL kanamycin at 37 °C and 200 rpm until an OD600 of 0.6 was reached. Protein expression was induced with 0.5 mM IPTG and 1 mM zinc sulfate, followed by incubation for 16 h at 18 °C. Cells were harvested by centrifugation and resuspended in lysis buffer (50 mM Tris–HCl, pH 8.5; 150 mM NaCl; 10 mM imidazole; 1 mM DTT). The lysate was prepared by sonication and clarified by centrifugation at 12,500 rpm for 40 min at 4 °C. The supernatant was purified via nickel-affinity chromatography using 5 mL of Ni-NTA resin as described,^15^ followed by size exclusion chromatography on a HighLoad Superdex 200 10/30 column equilibrated with 20 mM Tris, pH 7.4; 100 mM NaCl; 2 mM DTT. Pooled fractions were concentrated to 1.0 mg/mL, supplemented with 5% glycerol (v/v), flash-frozen in liquid nitrogen, and stored at −80 °C until use.

PL^pro^ enzymatic inhibition was assessed using the FRET substrate Abz-TLKGGAPIKEDDPS-EDDnp. Final assay concentrations were 70 nM enzyme and 27 μM substrate in buffer containing 50 mM HEPES pH 7.5, 0.01% Triton X-100 (v/v), and 5 mM DTT. Enzymes and compounds were preincubated at 37 °C for 10 min before substrate addition to initiate the reaction. For dose–response studies, inhibitors were titrated from 10 μM down to 0.6 pM and incubated under the same conditions. Fluorescence measurements (excitation/emission: 320/420 nm) were recorded every 30 s for 60 min at 37 °C using a SpectraMax Gemini EM Microplate Reader. Initial velocities were calculated from the linear portion of the reaction progress curves. Inhibition percentages were determined relative to a 1% dimethyl sulfoxide (DMSO) negative control. All assays were performed in technical triplicates, and IC_50_ values were derived from the averaged dose–response curves with corresponding deviations.

### 2.2 SARS-CoV-2 M^pro^ Cloning, Expression, Purification, and Activity Assays

The cloning, expression, and purification of M^pro^ have been described previously.^16,17^ In brief, recombinant plasmids were transformed into *E. coli* BL21 cells and cultured in ZYM 5052 medium until the culture reached an optical density at 600 nm (OD600) of 0.6. Protein expression was induced by lowering the temperature to 18 °C and incubating for 16 h. Cells were harvested by centrifugation at 5000*g* for 40 min at 4 °C and resuspended in lysis buffer (20 mM Tris, pH 7.8; 150 mM NaCl; 1 mM DTT). Cell disruption was achieved via sonication, and the lysate was clarified by centrifuging at 15,000*g* for 30 min at 4 °C. M^pro^ was obtained in its native form through autocleavage after expression. Protein purification began with ammonium sulfate precipitation at 1.5 M, followed by incubation on ice for 10 min. The precipitate was pelleted by centrifugation at 15,000*g* for 15 min at 4 °C, then resuspended in lysis buffer and applied to a Superdex 200 26/100 size exclusion chromatography column (GE Healthcare) equilibrated with gel filtration buffer (20 mM Tris, pH 7.8; 50 mM NaCl; 1 mM DTT). Further purification involved buffer exchange into 20 mM Tris, pH 8.0; 1 mM DTT and ion-exchange chromatography using a Mono-Q column (GE Healthcare), eluted with a linear gradient of 20 mM Tris, pH 8.0; 1 M NaCl; 1 mM DTT. Fractions containing pure protein were pooled and quantified by absorbance at 280 nm using a calculated extinction coefficient of 32,890 M^–1^ cm^–1^. Purity was verified by sodium dodecyl sulfate–polyacrylamide gel electrophoresis. For enzymatic assays, aliquots of purified protein at 0.5 mg/mL were flash-frozen in liquid nitrogen and stored at −80 °C.

M^pro^ enzymatic activity was measured using a FRET-based peptide substrate, DABCYL-KTSAVLQSGFRKM-E­(EDANS)-NH2, in an assay buffer containing 20 mM Tris (pH 7.3), 1 mM EDTA, and 1 mM DTT, with enzyme concentration at 140 nM. The enzyme was preincubated with test compounds in the assay buffer at 37 °C for 10 min before initiating the reaction by adding the substrate. The initial reaction velocity was determined from the linear portion of the fluorescence increase over time. Percent inhibition was calculated relative to a negative control containing 1% DMSO. Fluorescence readings were taken every 30 s for 60 min at excitation/emission wavelengths of 360/460 nm using a SpectraMax Gemini EM Microplate Reader at 37 °C.

### 2.3 HTS of Open Access Boxes

MB, PB, PRB, and CB were supplied by MMV,^2–4^ as part of the MMV Open program. Compounds were diluted to 1.0 mM prior to use in DMSO. For the screening, all compounds were tested in single doses at 10 μM, totaling 1% DMSO. Statistical calculations of *Z*-factor (*Z*′)^18^ were made as follows; *Z*′ = 1 – [3­(σ_p_ + σ_n_)/|μ_p_ – μ_n_|], where σ_p_ and σ_n_ are the standard deviation of positive (reaction without enzyme) and negative (reaction with enzyme) controls, respectively, and μ_p_ and μ_n_ are the means of positive and negative controls, respectively. For selected compounds that inhibited ≥80% of the enzyme activity, MMV provided resupply as solids to further evaluate the half-inhibitory concentration (IC_50_) and all follow-up experiments.

### 2.4 Molecular Docking

The PL^pro^ structure was obtained through the PDB code: 6WUU, and the protonation states of the amino acids were assigned with PDB 2PQR at pH 7.5.^19^ For the ligands, their p*K*a was checked with MolGpKa to assign their ionization states.^20^ Mol2 files of the inhibitors were generated using Omega.^21^ The compounds were docked using GOLD,^22^ with the binding pocket defined as all atoms within 12 Å away from the carbon alpha of residue Y268, a region where a similar compound is bound to PL^pro^ of SARS-CoV-1 (PDB code: 3MJ5). Pose analysis involved visual inspection for hydrogen bond formation with the D164 side chain and Y268 main chain.

### 2.5 Differential Scanning Fluorimetry

Differential scanning fluorimetry (DSF) assays were performed to compare the thermal stability of PL^pro^ with different compounds. The proteins, dyes, and compounds were diluted in assay buffer to a final concentration of 5 μM enzyme, 5× SPYPRO Orange (Invitrogen), and 20 μM each compound. 1% DMSO was used as negative control. The temperature increased from 25 to 75 °C in steps of 1 °C/min using a RT-PCR Mx3000P (Agilent) with the filter ROX (λex: 585 nm /λemi: 610 nm) to measure fluorescence intensity. The melting curves were fitted with a Boltzmann model using OriginLab9 software and the melting temperature obtained through the inflection point.

### 2.6 Preparation of Viral Stock

Clinical isolates of the SARS-CoV-2 Brazil/SPBR-02/2020 strain, obtained from patients confirmed positive for COVID-19 via RT-PCR, were propagated using Vero CCL-81 cell lines.^23^ To begin, Vero cells were maintained in Dulbecco’s modified Eagle’s medium (DMEM) supplemented with 10% heat-inactivated fetal bovine serum (FBS), along with penicillin (10,000 U/mL) and streptomycin (10,000 μg/mL), under standard incubation conditions of 37 °C with 5% CO_2_ and high humidity. For infection, a 1:100 dilution of the viral stock was introduced to the cultured cells and incubated for 48 h in FBS-free DMEM. This medium was supplemented with an antibiotic–antimycotic cocktail and TPCK-treated trypsin (1 μg/μL) to enhance viral entry into the host cells.

Following incubation, cytopathic effects (CPEs) were evaluated microscopically by using an inverted Olympus IX51 microscope. Cells displaying CPE were detached via scraping, collected, and centrifuged at 10,000*g* for 10 min at room temperature. Supernatants were then aliquoted and stored at −80 °C for future experiments. The viral titer was subsequently quantified on Vero CCL-81 cells using a standard limiting dilution method to determine the TCID_50_ (50% tissue culture infectious dose).

### 2.7 In Vitro SARS-CoV-2 Infection

In vitro evaluation of SARS-CoV-2 infection was carried out using three distinct cell lines: Vero CCL-81, Calu-3, and Caco-2. Cells were plated in 24-well plates at a density of 80,000 cells per well and cultured until approximately 90% confluency was achieved. The cells were then exposed to SARS-CoV-2 at a multiplicity of infection (MOI) of 1.0. Infections were performed using DMEM lacking FBS, supplemented with 1% antibiotic-antimycotic solution and trypsin-TPCK at a concentration of 1 μg/μL. After a 2 h incubation period, the viral inoculum was removed, and fresh medium containing varying concentrations of test compounds (125, 250, 500, and 1000 nM) or vehicle control (0.05% DMSO) was added. The cultures were then incubated for an additional 48 h at 37 °C in a 5% CO_2_ environment.

Images were captured with an IX51 inverted microscope to document the cellular morphology, and CPE in the Vero CCL-81 cells were analyzed using QCapture Pro 6.0 software (QImaging). Supernatants from infected cultures were harvested for RNA isolation, and viral RNA levels were measured via quantitative methods using a standard curve. All assays were performed in triplicates.

### 2.8 Cell Viability

The potential cytotoxic effects of the test compounds on Vero CCL-81, Calu-3, and Caco-2 cell lines were evaluated by using the Alamar Blue Cell Viability Assay (Thermo Scientific, Waltham, USA), following the protocol provided by the manufacturer. Fluorescence intensity was measured using the SpectraMax i-3 microplate reader (molecular devices), with excitation and emission wavelengths configured at 530 and 590 nm, respectively. Cell viability was expressed as a percentage, normalizing the average fluorescence of the untreated control cells to 100%. Viability under each treatment condition was then calculated relative to this baseline. All measurements were conducted in triplicates.

### 2.9 RT-PCR for SARS-CoV-2

Quantification of the SARS-CoV-2 genome was performed using primer-probe sets targeting the N2 region of the viral genome and the RNase-P housekeeping gene in accordance with protocols established by the U.S. CDC. Total nucleic acids were extracted from 250 μL of culture supernatant using the Trizol reagent (Invitrogen, CA, USA). For each reaction, 100 ng of RNA was combined with specific primers (20 μM), probe (5 μM), and TaqPath one-step qRT-PCR Master Mix (Applied Biosystems, Foster City, CA, USA) for one-step real-time RT-PCR amplification.

To determine viral load, a standard curve was generated using a plasmid containing a 944 bp insert from the N gene, beginning at nucleotide position 14. This cloned fragment—constructed using the PTZ57R/T CloneJetTM Cloning Kit (Thermo Fisher)—includes the target regions for all three CDC-designed primer/probe sets (N1, N2, and N3). Quantification was achieved using a 10-fold serial dilution of the plasmid, ranging from 10^6^ copies to a single copy per reaction.

### 2.10 Equipment for ADME Characterization

ADME profiling was conducted using liquid chromatography coupled with tandem mass spectrometry (LC–MS/MS). The chromatographic separation was carried out on a Prominence UFLC system (Shimadzu Corporation, Kyoto, Japan), connected to an LCMS-8045 triple quadrupole mass spectrometer (Shimadzu Corporation, Kyoto, Japan). The system was equipped with an electrospray ionization source for compound ionization during the detection.

### 2.11 Experimental Determination of Distribution Coefficient (eLogD7.4)

The estimation of the distribution coefficient (eLog*D*) was carried out using a chromatographic method based on the analyte retention times within a stationary phase. Separation was performed on a Supelco Ascentis Express RP Amide high-performance liquid chromatography (HPLC) column (5 cm × 2.1 mm, 2.7 μm particle size), utilizing a binary mobile phase system composed of 5% methanol in 10 mM ammonium acetate buffer at pH 7.4 (designated as solvent A), and pure methanol (solvent B). The gradient program was as follows: initial composition at 95% A; shifted to 100% A at 0.3 min; reduced to 0% A by 5.2 min; maintained at 0% A until 5.6 min; returned to 100% A at 5.8 min; and held until the end of the 7 min run. The injection volume for each sample was 5 μL.

Test compounds were diluted to a concentration of 1.0 mg/mL in a 1:1 mixture of mobile phases A and B, containing an internal standard at 200 nM. Final DMSO concentration was kept below 2%. To determine compound lipophilicity, each test molecule was injected individually along with a panel of eight reference drugs with known LogD values ranging from −1.86 to 6.10. These standards included: acyclovir (−1.86), atenolol (0.16), antipyrine (0.38), fluconazole (0.50), metoprolol (1.88), ketoconazole (3.83), tolnaftate (5.40), and amiodarone (6.10).^24–26^ A calibration curve was generated by plotting the retention times of these standards against their corresponding Log*D* values. The linear regression equation (*y* = *mx* + *b*) obtained from this curve was then used to calculate the experimental Log*D* (eLogD) of each test compound.

### 2.12 Human and Mouse Liver Microsomal Stability Assay

Metabolic stability of the test compounds was assessed using pooled human liver microsomes (20 mg/mL, GIBCO) and CD1 mouse liver microsomes (20 mg/mL, GIBCO). Compounds were diluted to a final concentration of 0.5 μM and incubated with microsomal protein at 0.25 mg/mL in phosphate-buffered saline (PBS) at pH 7.4. The DMSO content in the incubation mixture was maintained below 1%. The metabolic reaction was initiated by introducing NADPH as a cofactor at a concentration of 0.5 μM. Aliquots were taken at defined time intervals: 0 (immediately after NADPH addition), 5, 10, 20, 30, and 60 min. Reactions were halted by the addition of a quenching solvent consisting of a 1:1 mixture of acetonitrile and methanol containing an internal standard at 50 nM.

Following quenching, samples were centrifuged at 3500 rpm for 30 min to pellet the precipitated microsomal proteins. The resulting supernatants were analyzed via LC–MS/MS. Quantification was performed based on the peak area ratio (PAR) of the analyte to internal standard, with the signal at time zero defined as 100%. The percentage of parent compound remaining at each time point was calculated accordingly. Using the plot of % remaining versus incubation time, the degradation rate constant (*k*) was determined via nonlinear regression. From this, the half-life (*t*_1_/_2_ = ln(2)/*k*, in minutes) and intrinsic clearance (Clint = *k* × 1000/0.25, in μL/min/mg protein) were calculated.

Chromatographic analysis was carried out using a Supelco Ascentis Express C18 column (3 cm × 2.1 mm, 5 μm particle size). The mobile phases were composed of water with 0.1% formic acid (A) and acetonitrile with 0.1% formic acid (B). A binary gradient was applied as follows: 0–0.05 min, 95% A; 0.3:0.7 min, 2% A; 0.8:2.0 min, re-equilibration at 95% A. The total run time was 2 min per sample, with an injection volume of 10 μL and a flow rate of 0.7 mL/min. All metabolic stability assays were conducted in triplicates.

### 2.13 Parallel Artificial Membrane Permeability Assays

The passive permeability of the test compounds was evaluated using a 96-well parallel artificial membrane permeability assay (PAMPA) system (Corning Gentest, Cat. #353015). Working solutions were prepared by diluting compound stock solutions (10 mM) in PBS at pH 6.5 to a final test concentration of 10 μM, ensuring that the DMSO content remained below 1%. Each donor well received 300 μL of the compound solution, while the corresponding acceptor wells were filled with 200 μL of PBS at pH 7.4. The donor and acceptor plates were then assembled and incubated together at 37 °C for 5 h to allow for compound diffusion across the artificial membrane.

Aliquots from the initial donor solution (*T*_0_) were taken before incubation and stored at −20 °C. Upon completion of the incubation period, samples were collected from both the donor and acceptor wells. All samples, including *T*_0_ controls, were treated with a quenching mixture consisting of 10% water and 90% methanol/acetonitrile (1:1), containing 50 nM tolbutamide as the internal standard.

Quantification of compound concentrations in *T*_0_, donor, and acceptor wells was performed using LC–MS/MS. Chromatographic separation was carried out on a Supelco Ascentis Express C18 column (3 cm × 2.1 mm, 5 μm particle size). The mobile phases used were water with 0.1% formic acid (phase A) and acetonitrile with 0.1% formic acid (Phase B). A binary gradient elution was employed as follows: 0–0.05 min, 95% A; 0.3–0.7 min, 2% A; 0.8–2.0 min, re-equilibration at 95% A. The total runtime was 2 min per sample, with a flow rate of 0.7 mL/min and an injection volume of 10 μL.

Data obtained from the LC–MS/MS analysis were used to calculate the effective permeability coefficient (Pe) for each compound. All PAMPA experiments were performed in triplicate to ensure the reproducibility and accuracy of the results.

### 2.14 Kinetic Solubility

Kinetic solubility of the test compounds was assessed by preparing 10 mM stock solutions in DMSO, which were then dispensed into two 96-well incubation plates, each in duplicate. For each well, either PBS at pH 7.4 or 2.0 was added to reach a final compound concentration of 250 μM, keeping the DMSO content below 2.5%. The plates were sealed and incubated under shaking conditions (200 rpm) at 25 °C for 24 h to allow for equilibrium solubility to be reached.

Following incubation, any precipitated material was separated by centrifugation at 3000 rpm for 15 min at 25 °C. The resulting supernatants were subjected to LC–MS/MS analysis to quantify the soluble fraction of each compound.

To enable accurate quantification, a 0.5 mM intermediate standard was prepared by diluting the original 10 mM stock in a 1:1 solution of acetonitrile and water. Calibration curves were generated for each test compound and control by serial dilution of the intermediate standard to final concentrations of 50, 40, 20, 2, and 1 μM. The linear regression equation (*y* = *mx* + *b*) derived from each curve was used to calculate the actual concentrations present in the test samples.

LC–MS/MS analysis was carried out using a Supelco Ascentis Express C18 column (3 cm × 2.1 mm, 5 μm particle size). The mobile phase system consisted of water with 0.05% formic acid (A) and acetonitrile with 0.05% formic acid (B). A binary gradient elution was applied as follows: starting at 98% A; reaching 2% A at 1.2 min; held until 2.0 min; followed by a 0.6 min re-equilibration back to 98% A. The total run time was 2 min per sample, with an injection volume of 5 μL and a flow rate of 0.6 mL/min.

### 2.15 Plasma Stability

Plasma stability of the test compounds was assessed by using pooled K2EDTA-treated plasma obtained from female BALB/c mice. The plasma was diluted 1:1 with PBS, at pH 7.4, to prepare the working plasma solution. Test compounds were added to this solution in a 96-well incubation plate to achieve a final concentration of 2 μM. The plate was incubated at 37 °C with gentle shaking (50 rpm).

At designated time points (0, 60, 120, 240, and 360 min), 25 μL of samples were collected from each well and stored at −20 °C until further processing. Each sample was then mixed with 100 μL of a quench solution composed of acetonitrile and methanol (1:1, HPLC grade ≥ 99.9%), containing 200 ng/mL of an internal standard. The mixture was centrifuged at 4000 rpm for 10 min at 5 °C to precipitate plasma proteins. From each well, 50 μL of the resulting supernatant was transferred to a clean analytical plate for quantification.

Compound concentrations were measured using LC–MS/MS. Chromatographic separation was performed on a Supelco Ascentis Express C18 column (3 cm × 2.1 mm, 5 μm particle size). The mobile phases consisted of water with 0.1% formic acid (phase A) and acetonitrile with 0.1% formic acid (phase B). A binary gradient was applied as follows: 0 min −95% A; 0.3 min −2% A; 0.6 to 1.1 min −2% A; 1.2 min – return to 95% A; maintained at 95% A until 2.0 min. Each injection used a 10 μL volume, and the flow rate was set at 0.7 mL/min, with a total runtime of 2 min per sample.

The analyte-to-internal standard PAR was used to determine the compound stability. The PAR at time zero was set as 100%, and the percentage of compound remaining at each time point was calculated using the equation (% remaining = (PAR at time X/PAR at *T*_0_) × 100). All experiments were performed in triplicates.

### 2.16 Virus Generation

Antiviral cellular assays were conducted as previously outlined. Vero E6 cells (ATCC CRL-1586) were seeded into a T225 flask and cultured overnight at 37 °C in a 5% CO_2_ atmosphere using complete DMEM (Corning 15–013-CV) supplemented with 10% FBS, 1× penicillin–streptomycin (Corning 20–002-CL), and 2 mM l-glutamine (Corning 25–005-CL).

Following overnight incubation, the growth medium was removed, and 2 mL of the SARS-CoV-2 USA-WA1/2020 strain (BEI Resources NR-52281) diluted in complete DMEM was added at a MOI of 0.5. The cells were incubated for 30 min at 34 °C with 5% CO_2_ to allow virus adsorption. Subsequently, 30 mL of complete DMEM was added to the flask, which was then maintained at 34 °C with 5% CO_2_ for an additional 5 days.

On the fifth day postinfection, the culture supernatant was collected and clarified by centrifugation at 1000*g* for 5 min. The clarified supernatant was passed through a 0.22 μm filter to remove cellular debris and then stored at −80 °C until further use.

### 2.17 HeLa-ACE2 Stable Cell Line

HeLa-ACE2 cells were produced by transducing HeLa cells with a lentivirus encoding human ACE2, enabling infection by SARS-CoV-2.^28^ The lentiviral particles were generated by cotransfecting HEK293T cells with the pBOB-hACE2 plasmid alongside packaging plasmids pMDL, pREV, and pVSV-G (sourced from Addgene) using Lipofectamine 2000 (Thermo Fisher Scientific, Cat. No. 11668019).

48 h post-transfection, the viral-containing supernatant was harvested and used to infect preplated HeLa cells. After 12 h of exposure, transduced cells were collected, expanded, and cryopreserved to establish stable ACE2-expressing cell lines.

The cells were cultured in DMEM (Gibco, 11965-092) supplemented with 10% FBS (Gibco, 10438026) and 1× sodium pyruvate (Gibco, 11360070) at 37 °C in a humidified atmosphere with 5% CO_2_.

### 2.18 SARS-CoV-2/HeLa-ACE2 High-Content Screening Assay

Compounds were dispensed acoustically into 384-well μClear-bottom plates (Greiner, part no. 781090-2B). HeLa-ACE2 cells were seeded at 1.0 × 10^3^ cells per well in 13 μL of DMEM containing 2% FBS. Plates were then transferred to the BSL-3 laboratory, where 13 μL of SARS-CoV-2, diluted in assay medium, was added to each well at a MOI of 2.2 for initial screening and adjusted to 0.65 for powder reconfirmation assays.

Following virus addition, plates were incubated for 24 h at 34 °C in a 5% CO_2_ atmosphere. Cells were then fixed using 4% formaldehyde for 1 h at 34 °C with 5% CO_2_. Between fixation and antibody staining steps, plates were washed with 1× PBS containing 0.05% Tween 20.

Primary staining involved adding human polyclonal plasma (a limited, unique reagent supplied in 500 μL aliquots) diluted 1:500 in perm/wash buffer (BD Biosciences 554723), incubated at room temperature for 2 h. This was followed by incubation with a mixture of goat antihuman H + L Alexa Fluor 488 conjugate (8 μg/mL, 1:250 dilution; Thermo Fisher Scientific A11013) and 3 μM DAPI (Thermo Fisher Scientific D1306) in SuperBlock T20 (PBS) buffer (Thermo Fisher Scientific 37515) for 1 h at room temperature in the dark.

Imaging was performed on the ImageXpress Micro Confocal High-Content Imaging System (Molecular Devices) using a 10× objective, capturing four fields per well. Image analysis employed the Multi-Wavelength Cell Scoring module in MetaXpress software, where DAPI staining identified cell nuclei, quantifying total cell numbers, and SARS-CoV-2 immunofluorescence marked infected cells. Compounds exhibiting a selectivity index (SI = CC_50_/EC_50_) greater than 3 were classified as active.

### 2.19 Uninfected Host Cell Cytotoxicity Counter Screens

Compounds were acoustically dispensed into 1536-well μClear plates (Greiner, part no. 789091). HeLa-ACE2 cells, maintained as described in the infection assay, were seeded into these assay-ready plates at a density of 400 cells per well in DMEM supplemented with 2% FBS. Plates were incubated for 24 h at 37 °C with 5% CO_2_.

Cell viability was evaluated using the Image-iT DEAD Green reagent (Thermo Fisher), following the manufacturer’s protocol. Subsequently, cells were fixed with 4% paraformaldehyde and counterstained with DAPI. Imaging was performed on the ImageXpress Micro Confocal High-Content Imaging System (Molecular Devices) using a 10× objective. Quantification of total live cells per well was carried out with the Live Dead Application Module in MetaXpress software based on the acquired images.

### 2.20 Data Analysis

High-content image data analysis was performed by using MetaXpress software (version 6.5.4.532). Results from the primary in vitro screen and host cell cytotoxicity counter screens were imported into Genedata Screener, Version 16.0.3-Standard, for processing. Data from HeLa-ACE2 assays were normalized against neutral controls (DMSO) and inhibitor controls (2.4 μM remdesivir for antiviral activity and 9.6 μM puromycin for host cell toxicity in infected cells). For Calu-3 infection assays, normalization was done using neutral (DMSO) and inhibitor control (10 μM remdesivir), while cell count readouts were normalized between the stimulator (10 μM remdesivir) and neutral controls. In cytotoxicity counter assays with uninfected cells, positive inhibitory controls were 40 μM puromycin for HeLa-ACE2 cells and 30 μM puromycin for Calu-3 cells (Sigma). Dose–response testing of compounds was conducted in technical triplicate across multiple assay plates, with dose–response curves fitted using the four-parameter Hill equation. Median condensing of technical replicate data was applied as previously outlined.^27^

## 3 Results

### 3.1 Screening of Open-Access Libraries Identifies SARS-CoV-2 PL^pro^ Inhibitors

To identify potential inhibitors of SARS-CoV-2 proteases, three open-access libraries from MMV and one from MMV/DNDi were screened using end point assays at a final concentration of 10 μM. No compounds from these collections inhibited M^pro^ activity by ≥ 80% (Figure 1a). However, four compounds—MMV1579849 (P-113D), MMV1634402 (Brilacidin), MMV002459 (Tobramycin), and MMV1634397—exhibited ≥80% inhibition of SARS-CoV-2 PL^pro^. These hits were further analyzed in dose–response assays, yielding IC_50_ values of 14.79, 6.93, 4.16, and 0.7 μM, respectively (Figure 1b).

**Figure 1:**
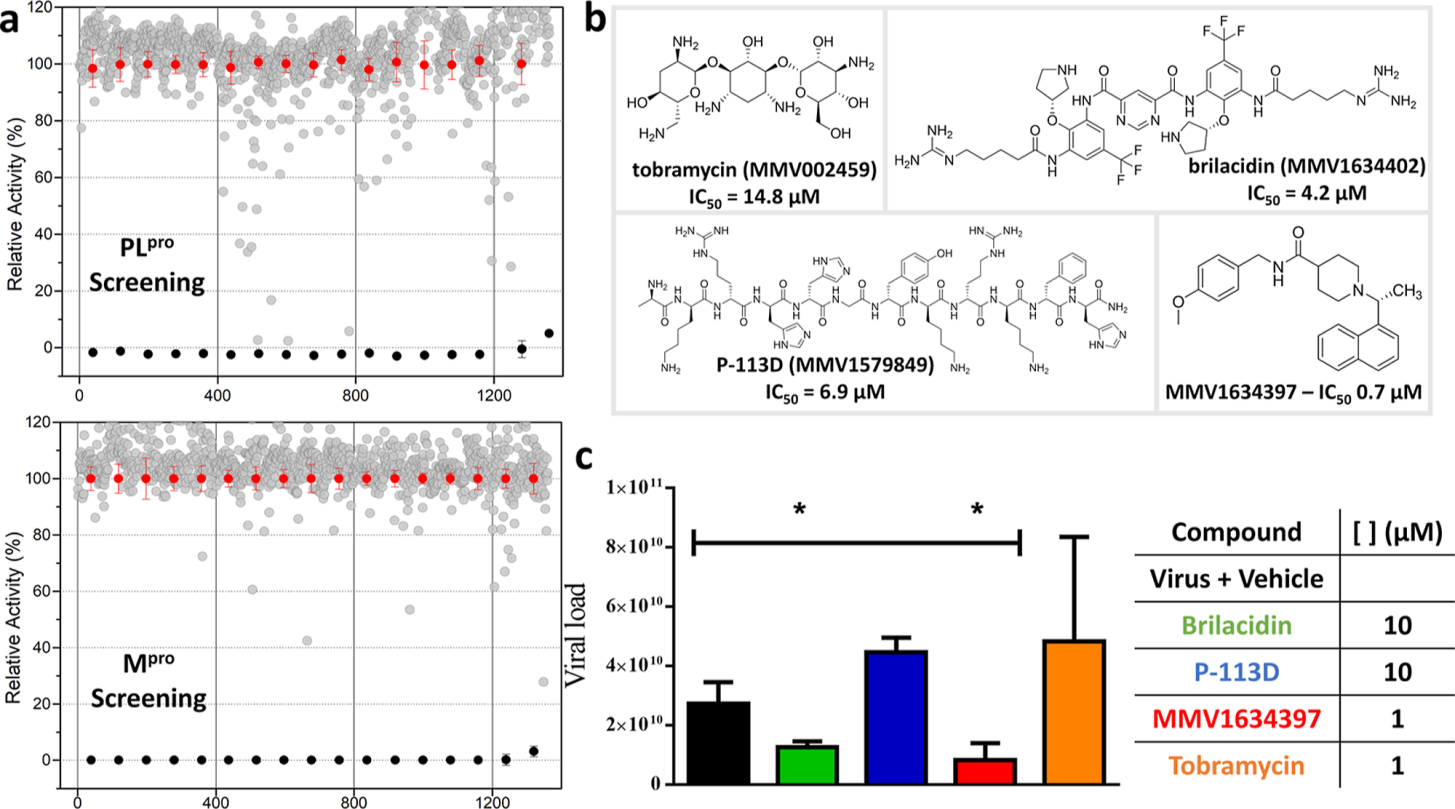
Summary of screening results for MMV Open Boxes against SARS-CoV-2 M^pro^ and PL^pro^. (a) Scatter plots showing screening results for PL^pro^ (top) and M^pro^ (bottom) against the MMV open boxes compound collection. The *y*-axis represents the relative enzymatic activity in the presence of tested compounds. Compounds were screened at 10 μM (gray) and compared to the DMSO control (red) and a no-enzyme control (black). (b) List of MMV open boxes hits identified for PL^pro^, along with their respective IC_50_ values (μM). (c) Antiviral activity of selected hits in Vero E6 cells infected with SARS-CoV-2, tested at 10 μM or 1 μM.

### 3.2 Antiviral Screening of the Open Boxes Selected Compounds

Primary evaluation of antiviral activity of these compounds was assessed in vitro using SARS-CoV-2-infected Vero CCL-81 cells in doses of 1 and 10 μM. Viral load reduction, measured by RT-PCR, revealed that MMV1634397 and Brilacidin decreased viral replication by over 50% at 10 and 1 μM, respectively (Figure 1c). Given its potency in both enzymatic and cell-based assays, MMV1634397 was selected for further optimization.

### 3.3 Structure–Activity Relationship and Molecular Docking of MMV1634397 Analogs

Eighteen analogs of MMV1634397 were synthesized by TCG to investigate its structure–activity relationship (SAR), summarized in Table 1, and details of synthesis are presented in SMI. Figure 2 summarizes the five key regions of the scaffold analyzed and their corresponding IC_50_ values, with stereochemistry inferred from the activity in PL^pro^. Overall, most substitutions led to decreased activity from the reference compound MMV1634397 which exhibited an IC_50_ of 0.70 ± 0.05 μM. Notably, modifications in the naphthyl moiety (**2**, **3**, **14**), deletion of the C5 methyl group (**1**), or amide bond inversion (**6**, **7**, **8**, **9**) resulted in a significant or complete loss of inhibition, with IC_50_ values increasing to 22 μM or higher. Therefore, these regions are highly sensitive to changes in the size or bond orientation. Conversely, some modifications were well tolerated or even improved activity. Replacing the piperidine moiety with pyrrolidine retained potency with an IC_50_ of 10 μM and 1.8 μM for the S–R (**15**) and S–S (**17**) enantiomers, respectively. However, chirality of the C5 methyl was essential for activity of these compounds as both R–S (**16**) and R–R (**18**) enantiomer were inactive with an IC_50_ higher than 200 μM. The most substantial potency enhancement was observed with substitutions in the benzyl ring, where *p*-Cl (**13**), > *p*-CH_3_ (**5**) > pyridine (**10**) > cyclohexane (**11**) ranked as the most favorable replacements in order of efficacy with IC_50_ of 0.06, 0.16, 0.34, and 1.2 μM, respectively. Similar to the previous results, the C5 methyl group is preferred in the S conformation, with the R enantiomer of *p*-CH_3_ (**4**) and *p*-Cl (**12**) showing a lower IC_50_ of 0.52 and 1.25 μM, respectively.

**Table 1:** Inhibition and Thermal Stability of PL^pro^ in the Presence of MMV1634397 and Analogs^a^

compound number	MMV code	PL^pro^ IC_50_ (μM)	Δ*T*_m_ (°C)
**1**	MMV1848784	22 ± 3	0.40 ± 0.08
**2**	MMV1898892	>20	1.50 ± 0.05
**3**	MMV1898893	28 ± 4	0.06 ± 0.05
**4**	MMV1898894	0.5 ± 0.2	7.9 ± 0.6
**5**	MMV1898895	0.161 ± 0.005	5.57 ± 0.09
**6**	MMV1898896	>20	4.8 ± 0.1
**7**	MMV1898897	>20	–0.30 ± 0.05
**8**	MMV1898898	>200	0.28 ± 0.05
**9**	MMV1898899	>200	0.50 ± 0.05
**10**	MMV1898900	0.34 ± 0.06	4.6 ± 0.1
**11**	MMV1898901	1.2 ± 0.2	0.90 ± 0.07
**12**	MMV1898902	1.2 ± 0.3	6.6 ± 0.2
**13**	MMV1898903	0.06 ± 0.05	6.1 ± 0.4
**14**	MMV1898913	>200	1.20 ± 0.06
**15**	MMV1899571	10 ± 2	0.40 ± 0.06
**16**	MMV1899572	>200	0.40 ± 0.08
**17**	MMV1899573	1.80 ± 0.05	–1.5 ± 0.1
**18**	MMV1899574	>200	–1.0 ± 0.1
**reference**	MMV1634397	0.70 ± 0.05	6.3 ± 0.3

a The IC_50_ values (average of triplicates with SD, in μM) indicate the concentration required to inhibit 50% of PL^pro^ activity, while the thermal shift (Δ*T*_m_ as an average of triplicates with SD, in °C) reflects changes in protein stability upon ligand binding. The reference compound (MMV1634397) is included for comparison.

**Figure 2:**
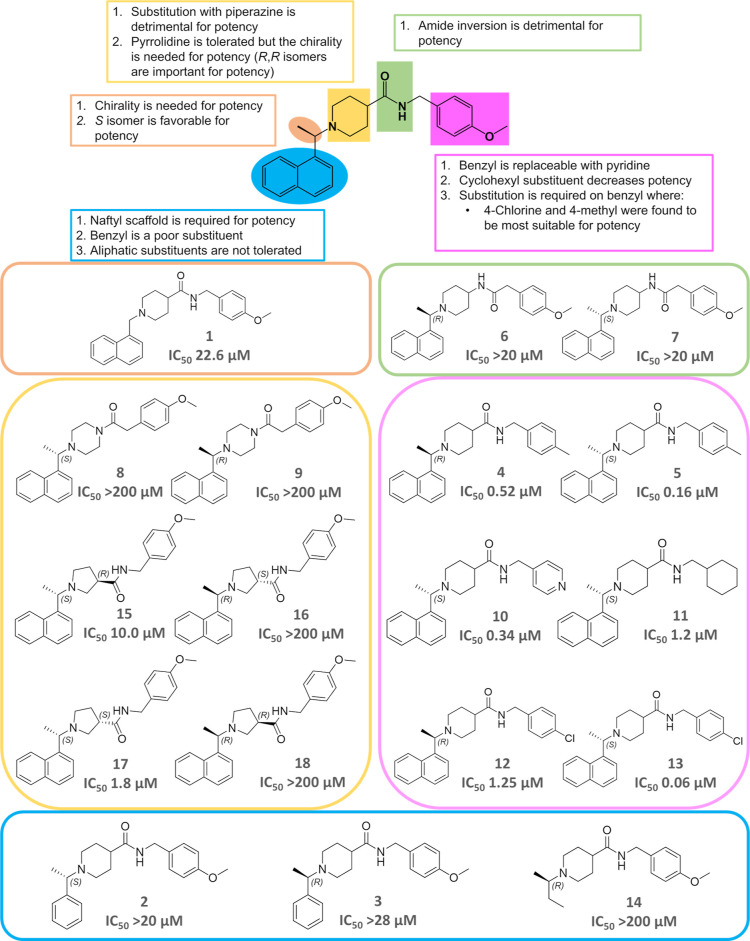
SAR panel for MMV1634397 analogs. Compound number and their IC_50_ values for PL^pro^ are written below each compound.

Since this class of compounds is known to target the active site of the enzyme,^29^ molecular docking studies were performed to rationalize the binding mode of MMV1634397 analogs. Docking results show that the replacement of the six-membered piperidine ring (as in compound **13**, Figure 3a) with a five-membered pyrrolidine ring of the preferred stereochemistry (as in compound **17**, Figure 3b) maintains a comparable ligand orientation within the PL^pro^-binding site and competes with the peptide-binding site (Figures 3c and S3). A key hydrogen bond is formed between the ligand’s amide group and the main-chain carbonyl of Y268, which likely explains why inversion of the amide bond leads to a complete loss of activity, such inversion disrupts the hydrogen bonding interaction. The piperidine or pyrrolidine nitrogen, which is expected to be protonated at pH 7.5, forms an electrostatic interaction with the negatively charged side chain of D164. This interaction tolerates ring size but is highly sensitive to stereochemistry. Changing the stereochemistry of the pyrrolidine ring disrupts the amide–Y268 hydrogen bond and causes steric clashes with the BL2 loop, consistent with docking results showing no favorable poses for these isomers.

**Figure 3:**
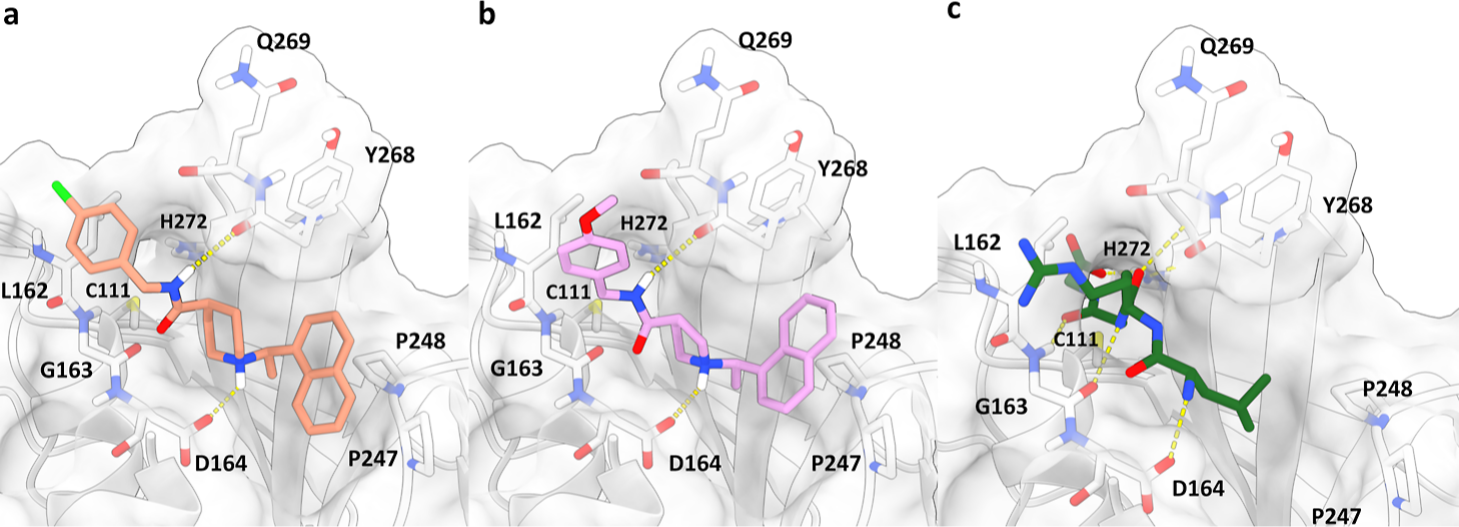
Predicted binding modes for selected compounds bound to SARS-CoV-2 PL^pro^. (a) Compound 13 (orange). (b) Compound 17 (pink). (c) Peptide LRGG (green) from the ubiquitin structure in complex with SARS PL^pro^ (PDB id: 4MOW). Key residues are depicted as sticks. Key hydrogen bonds are depicted as yellow dashed lines.

Although substitutions on the benzyl ring do not appear to make crucial contacts with the protein, the aromatic moiety is positioned near the peptide backbones of L162, G163, and D164 and may engage in π-stacking interactions. Among these substitutions, compounds bearing *p*-Cl and *p*-CH_3_ substituents showed greater potency than the pyridyl analog. This trend is not explained by protein interactions but may be driven by solvation/desolvation effects. Experimental hydration free energy values suggest that pyridine has a much higher desolvation penalty (−4.69 kcal/mol) compared to *p*-chlorobenzyl (−1.12 kcal/mol) and *p*-methylbenzyl (−0.90 kcal/mol),^30^ making it energetically less favorable to transfer from water into the hydrophobic binding site. Thus, the entropic component of binding is likely to contribute to the observed differences in potency across the benzyl ring substitutions.

### 3.4 Thermal Stability Analysis of PL^pro^ in the Presence of MMV1634397 Analogs

The thermal stability of PL^pro^ was evaluated by DSF in the presence of MMV1634397 and all of its analogs. The compounds tested exhibited a wide range of values for variation in melting temperature (Δ*T*_m_). Analogs with substitutions in the phenyl ring showed the highest temperature shifts such as *p*-CH_3_ (**4**) with 7.98 °C, followed by both enantiomers of *p*-Cl (**12**, **13**) 6.6 and 6.15 °C, and MMV1634397 with 6.26 °C. Other notable substitutions with relatively high temperatures included the S enantiomer of *p*-CH_3_ (**5**) 5.57 °C, amide inverted (**6**) 4.8 °C, and pyridine containing (**10**) 4.65 °C. In contrast, some compounds displayed negative temperatures, such as the R isomer of the inverted amide (**7**) −0.3 °C and both S–S and R–R isomers of the pyrrolidine-substituted compounds (**17**, **18**) −1.5 °C and −1 °C, respectively. The remaining compounds had lower positive temperatures, ranging from 0.06 °C (**3**) to 1.5 °C (**2**) (Figure S2).

As expected, only analogs with substitutions in the benzyl ring exhibited similar or higher melting temperatures compared to the original compound (Δ*T*_m_ = 5.57–8 °C). In contrast, other analogs showed reduced or negligible effects on both potency and thermal stability, highlighting the critical role of the benzyl ring region in maintaining the binding interaction. Interestingly, a clear correlation between pIC_50_ and Δ*T*_m_ was observed (*R* ^2^ = 0.49), showing the potential of DSF as a biophysical tool for evaluating PL^pro^ inhibitors ranging from modest to high potency (Figure 4).

**Figure 4:**
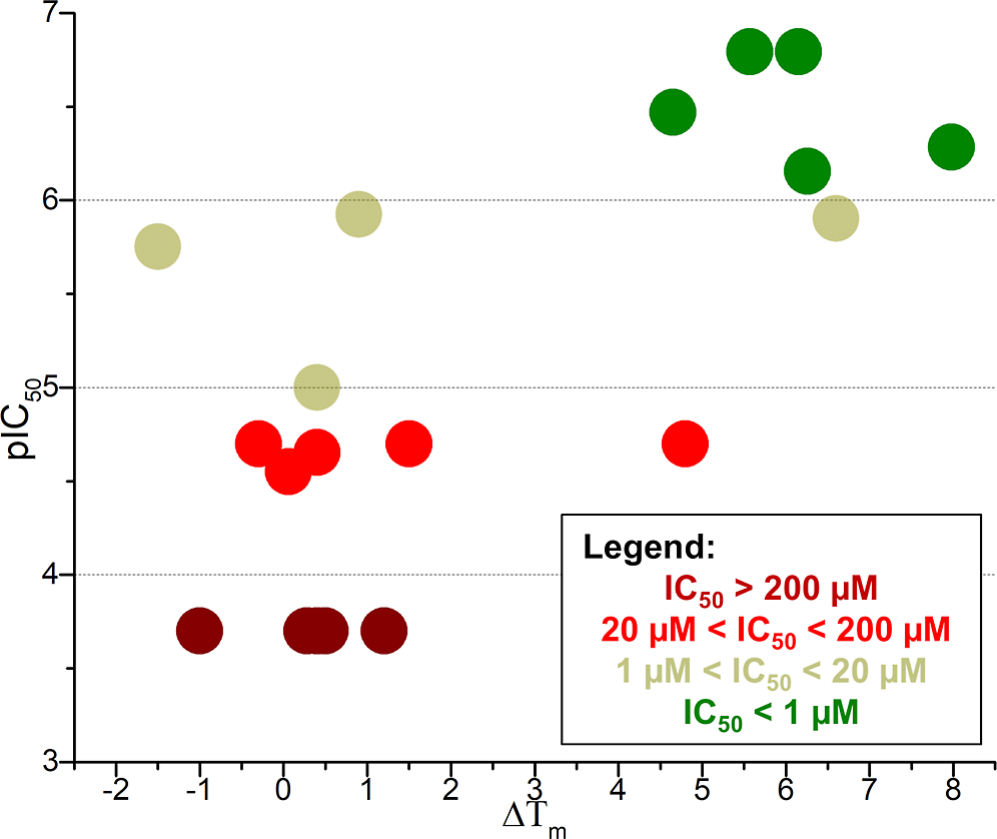
Plot depicts the correlation between compound potency and thermal stabilization of SARS-CoV-2 PL^pro^. The *y*-axis represents the pIC_50_ values (−log *IC*_50_, M), while the *x*-axis shows the thermal shift (Δ*T*_m_, in °C). Compounds are color-coded based on their potency: the most potent inhibitors are highlighted in green, followed by beige, red, and dark red for less active compounds.

### 3.5 Cellular Activity of Best Analogs

Based on potency and thermal stability, the analogs with benzyl ring substitutions (**4**, **5**, **10–13**) and the most potent pyrrolidine ring (**17**) were further analyzed with cell-based assays using HeLa ACE2 cell-lines. Only compounds with *p*-Cl substitution (**12**, **13**) or a pyrrolidine ring (**17**) reached more than 50% inhibition in the highest concentration tested (9.61 μM) and had their EC_50_ calculated (Table 2). Both enantiomers of *p*-Cl were the most potent compounds, with the R isomer (**12**) (EC_50_ = 2.9 μM, CC_50_ = 14.4 μM, S.I = 5.0) being slightly more potent than S (**13**) (EC_50_ = 3.7 μM, CC_50_ = 18.8 μM, S.I = 5.0). The pyrrolidine replacement (**17**) showed the lowest potency with an EC_50_ of 6.4 μM (CC_50_ = 29.6 μM, S.I. = 5.0). Details and curves are listed in Table S1 and in Figure S4. Despite the potency of the compounds, their S.I. are small, mainly due to cytotoxicity of the compounds. Future optimizations are needed to increase their effectiveness by either improving the potency or by decreasing their toxicity.

**Table 2:** Cellular Activity of Selected MMV1634397 Analogs against SARS-CoV-2 in HeLa-ACE2 Cells (Average of Triplicates)

compound	SARS-CoV-2 EC_50_ [μM]	uninfected HeLa-ACE2 CC_50_ [μM]	SI (average CC_50_/average EC_50_)
**4**	>9.61	>39.8	NA
**5**	>9.61	>39.8	NA
**10**	>9.61	>39.8	NA
**11**	>9.61	>39.8	NA
**12**	2.9 ± 0.2	14.4 ± 0.2	4.8
**13**	3.7 ± 0.5	18.8 ± 0.2	5.0
**17**	6.4 ± 0.4	29.6 ± 0.2	4.5

### 3.6 Early ADME Profiling of MMV1634397 and Analogs

One of the reasons for the high attrition rate in drug discovery is the lack of good pharmacokinetic and ADME properties after lead optimization phase.^31^ The focus exclusively on increasing potency discards compounds that could possess similar activity but improved pharmacokinetics properties. In this regard ADME profiling early on lead optimization phase is being extensively used to decrease failure of drug candidates in posterior phases, speeding up the development process.^32^ An in vitro ADME profiling was performed to evaluate the pharmacokinetics properties of the MMV1634397 analogs (Table S2).

Kinetic solubility was assessed, and most compounds exhibited high solubility, with values ranging from >20 to 30 μg/mL, while more lipophilic compounds (**4**, **5**, **11**) showed lower solubility, ranging from 6 to 8 μg/mL. The partition coefficients were similar, with an eLog*D* of 4–4.8, near the threshold of the Lipinski Rule of 5, which is commonly used to assess drug likeness and oral bioavailability. The partition coefficient is indicative of a compound’s ability to cross lipid membranes, with values above 5 suggesting limited permeation (Table 3).

**Table 3:** Summary of Biochemical Inhibition, Antiviral Activity, Cytotoxicity, and ADME Properties for Top Compounds^a^

compound		**4**	**5**	**10**	**13**	**17**
IC_50_ (μM)		0.52	0.16	0.34	0.06	1.8
HeLA-ACE2		>9.61	>9.61	>9.61	3.7	6.7
EC_50_ (μM)						
HeLa-ACE2		>39.8	>39.8	>39.8	18.8	29.6
CC_50_ (μM)						
eLogD		4.56	4.55	3.32	4.70	4.25
pH 7.4						
Pe (10^–6^ cm/s)		0.78	0.67	1.01	0.30	0.61
pH 6.5–7.4						
kinetic solubility	pH 2.0 (μg/mL)	>24,21	>25,03	>17,28	>24,36	>24,03
	pH 7.4 (μg/mL)	8.09	8.62	>30.98	>13.38	>19.88
HLM	*T*_1/2_ (min)	35.36	18.24	36.67	15.07	21.73
	CL_int_(mic) (μL/min/mg)	78.40	152.00	75.60	184.00	127.60
MLM	*T*_1/2_ (min)	12.96	9.48	25.30	9.19	10.27
	CL_int_ (mic) (μL/min/mg)	214.00	292.40	109.60	301.60	270
plasma stability	remaining 60 min (%)	58.47	33.79	97.19	46.83	73.01
	*T*_1/2_ (min)	385.08	239.02		364.81	770.16

a Permeability (Pe), human liver microsome (HLM), mouse liver microsome (MLM).

Permeability assays revealed that the initial hit compound had a permeability of 1.07 × 10^–6^ cm/s, classifying it as a medium to low permeable molecule. Most analogs showed similar or lower values, with some compounds lacking the naphthalene moiety (**3**) and with amide inversion (**6**) being highly permeable. Interestingly, the most potent compounds in the series exhibited the lowest permeability, suggesting that despite potency improvements, their efficacy in vitro and in vivo could be limited by lower bioavailability.

Metabolic stability was assessed using mouse and human liver microsomes to measure the compound clearance and half-life. For the initial compound, the clearance and half-life in human liver microsomes were 80 μL/min/mg and 35 min, respectively, classifying it as moderately stable. Most analogs displayed similar or lower metabolic stability, except analogs lacking the naphthalene moiety (**2**, **3**, **14**), which showed improved stability (Table 3).

In summary, the early ADME profile shows moderate or poor initial pharmacokinetics for some analogs, with areas where optimization is required. The permeability and stability are parameters related to drug absorption and bioavailability, which are key to achieving effective activity in vivo. In most analogs, especially the most active molecules (4, 5, 12, 13), these parameters were moderate to low, suggesting that optimization is strictly required to guarantee better absorption and bioavailability (Table 3). On the other hand, the kinetic solubility of most compounds was high, indicating that increasing the dosage to achieve a higher bioavailability might not be an issue.

The optimization of the compounds can be guided by an interesting pattern seen in the ADME profile, regarding chirality and metabolic stability. Analogs with *R*-chirality exhibited better clearance and half-life compared to their *S*-isomer, with a 2-fold difference in most cases (Table S2). This suggests that while chirality is not essential for potency, it can enhance metabolic stability. Additionally, replacing the naphthyl moiety with a methyl group resulted in a 10-fold increase in half-life, though none of these compounds showed inhibitory activity.

## 4 Discussion

Despite the clinical approval of several M^pro^ inhibitors^11,33^ and the late-stage development of many others,^13,34–36^ no PL^pro^ inhibitor has yet advanced to clinical use. PL^pro^ plays a crucial role in SARS-CoV-2 replication and immune evasion, making it an attractive therapeutic target. However, the development of PL^pro^ inhibitors has been challenging due to their broad substrate specificity and the structural flexibility of their active site, which hinders the design of selective and potent compounds. Yet, novel PL^pro^ inhibitors have already demonstrated significant potential in inhibiting viral infections, including through oral administration.^37,38^ Therefore, identifying inhibitors that effectively bind and stabilize PL^pro^ while maintaining antiviral activity in cellular models remains important, specifically with the rise of resistance to M^pro^ inhibitors and the potential of using combinatory drug treatment aiming at different modes of actions.

Different target-based studies on MMV Open box libraries were previously reported, with most studies focusing on virtual screening campaigns targeting M^pro^.^39–41^ The best compounds identified through these studies did not reach an inhibition of >80% at 10 μM in our testing. Instead, we found **MMV1634397** as a promising hit for the inhibition of PL^pro^ activity and performed an initial SAR and ADME profiling of its synthesized analogs. The high activity is not unexpected as MMV1634397 belongs to the class of chalcone-amides inhibitors. This class has been extensively studied and optimized for both SARS-CoV-1 and 2, usually using GRL0617 as a starting scaffold.^42^ The hit compound was first mentioned in an SAR optimization study performed by Ghosh et al., where different analogs were evaluated for inhibition of SARS-CoV-1 PL^pro^.^43^ A second SAR study was later performed by Báez-Santos et al., where besides inhibition in SARS-CoV-1 PL^pro^, the metabolic stability was also assessed.^44^ Interestingly, only a few of those analogs were evaluated in SARS-CoV-2, showing similar inhibition and binding mode.^45^

In this context, our results corroborate with previous SARS-CoV-1 data or give new insights into the chalcone-amide class of compounds. For instance, the requirement of a methyl group in position C5 for activity (hit vs **1**), and that its chirality does not severely affect the potency, was already noticed in the first SAR performed for SARS-CoV-1.^43^ The same holds true for the loss of activity when the amide bond is inverted or when a piperazine group is added instead of a piperidine.^43,46^ However, some analogs had different modifications not studied before. It is known from X-ray structures in complex with similar compounds in both SARS-CoV-1 and 2 that the benzyl moiety is placed close to Q269 and Y268, which are in the BL2 loop, a loop that closes the compound in its active position.^43,45^ However, mutational studies have shown that alterations at Q269 do not significantly affect inhibitor potency, indicating that this side chain does not form strong interactions with the benzyl ring or its substituents.^44^ This can be noticed as the analogs we have tested with a methyl group have comparable potency to polar or halogen groups. Consistent with these findings, our docking studies revealed no specific interaction between the benzyl ring and the side chain of Q269. Instead, the amide group of the ligands forms a key hydrogen bond with the backbone of Y268. When the amide group of the ligand is inverted, this interaction is disrupted and the compound loses potency. Conversely, the π-conjugated system of the benzyl ring may undergo π-stacking interactions with the amide groups from the main chain of L162, G163, and D164, and this interaction could explain the decreased potency observed when a nonaromatic ring is used instead. Additionally, modifications in the benzyl moiety, such as *p*-Cl and *p*-Me, or changing it to a pyridine ring impact the desolvation energy of the ligand, which can be correlated with their relative potency. For instance, the pyridine analogue exhibits a more negative hydration free energy and lower potency than the less polar *p*-Cl and *p*-CH_3_ derivatives. These observations suggest that changes in polarity at this position affect the ligand’s desolvation penalty upon binding, thereby influencing overall binding affinity. This supports the hypothesis that the benzyl substituent primarily contributes via π-stacking interactions with the backbone residues (L162, G163, and D164), while polarity-driven desolvation effects further modulate binding potency.^44^

Finally, we also evaluated the effect of ring size for activity by replacing the piperidine with pyrrolidine. Interestingly, the ring change makes the compound sensible to the chirality of the methyl group, with the S enantiomer being inactive, similar to what is seen in GRL0617.^47^ The loss of chirality freedom probably comes from the smaller ring conformation, that cannot account the right positioning of the methyl group for both chirality’s, as it happens with the piperidine ring. Nevertheless, the compound was still active in cell-based assays but with a lower potency of EC_50_: 6.8 μM, showing that a smaller ring can also maintain cellular activity.

The most potent compound (**13**) inhibited SARS-CoV-2 viral replication in HeLa cells, with an EC_50_: 3.7 μM and S.I: 5. Despite their antiviral activity, future optimizations are required to decrease the compound toxicity, and different halogen or nonhalogen groups should be evaluated to see whether the potency can be improved without increasing toxicity. For example, the use of *p*- or *m*-fluorine, *p*-ethyl and *p*- or *m*-methyl carboxamides increased potency but not toxicity in a cell-based assay for SARS-CoV-1 infections.^44^ These substitutions can be used as a starting point for future optimizations to decrease the cytotoxicity.

The results showed that the compounds have a promising ADME profile with only permeability and metabolism stability showing moderate or low values. The substitutions in the benzyl ring were the most critical for permeability, reaching a decrease of 4-fold in the most potent compound (Table 3). The lower permeability after the substitutions can explain a common problem seen in most of the SAR done with this class of compounds, where even though the potency of the compounds is increased, their cellular activity is not improved in a similar manner.^43–45^

The metabolic stability can be used as a predictor of drug bioavailability and half-life due to the importance of these parameters for in vivo activity, optimization early on the drug discovery process helps to decrease the attrition rate in the later stages.^48^ Our data show that this class of compounds has a moderate metabolism stability in human or mouse liver microsomes; however, most substitutions that increased potency decreased or did not optimize the stability. In human liver microsomes, naphthalene is converted to *trans*-1,2-dihydrodiol and 1-naphthol, which can impact the metabolism of compounds containing naphthalene.^49^ The large increase in stability of analogs without a naphthyl group further confirms that the presence of this group is responsible for a moderate/low metabolism stability. Since the binding pocket of the naphthalene group is the same as GRL0617, we can use the extensive SAR studies done with this compound to suggest groups that can be used to replace the naphthalene ring without decreasing its potency.^43^ One of these studies was performed by Shen et al., where using GRL0617 as the starting hit, they explored different pockets available for growing the compound. It was shown that the naphthalene can be replaced with a 2-phenylthiophene improving both potency and metabolism stability in human liver microsomes.^50^

The cellular antiviral activity seen by the most potent compounds (IC_50_: EC50:3 μM) together with the initial ADME profiling shows that the chalcone-amides class of compounds are promising for the development of new drug candidates for SARS-CoV-2 targeting PL^pro^. The nanomolar potency of the best compounds is equivalent to most inhibitors recently developed for PLpro.^37,51–54^ However, optimization for improving toxicity and metabolism stability is essential since most of the latest inhibitors reached ideal drug-like properties during hit development. The metabolic stability can be improved with naphthyl substitutions as previously discussed, whereas for cellular activity, new substitutions are needed considering the permeability and cytotoxicity of the compound. Past SAR data of the same compound or GLR0617 analogs can be used to further guide and avoid pitfalls that were already explored, increasing the success of future optimization campaigns. Finally, our study highlights the potential of screening MMV Open Boxes in target-based campaigns to identify promising scaffolds for drug development.

## Supplementary Material







## Notes

Table S1 - Detailed antiviral data of compounds (XLSX)
Table S2 - Detailed PK data of compounds (XLSX)
Chemical synthesis details and Figures S1–S4 (PDF)
