# Supplementary material for: Screening of Medicines for Malaria Venture Open Boxes Identifies Potent SARS-CoV‑2 Papain-like Protease (PLpro) Inhibitors
Source: ACS Omega. 2025 Sep 11;10(37):42711–24. doi: 10.1021/acsomega.5c04858 (PMC12461331; doi:10.1021/acsomega.5c04858)
Supplement: Supplementary file 3 [file ao5c04858_si_003.pdf]

Supplementary Information

Screening of Medicines for Malaria Venture Open Boxes Identifies Potent SARS-CoV-2 Papain-like Protease (PL^{pro}) Inhibitors

Victor Oliveira Gawriljuk[1], Gabriela Dias Noske[1], Rafaela Sachetto Fernandes[1], Aline Minalli Nakamura[1], Marjorie C. L. C. Freire[1], Mariana Ortiz Godoy[1], Vinicius Bonatto[1], Rafael Chelucci[1], Adriano Andricopulo[1], Malina A. Bakowski[2], Karen C. Wolff[2], Laura Riva[2], Jeremy N. Burrows[3], Timothy N. C. Wells[3], Benoît Laleu[3], Ronaldo Martins[4,5], Juliano Paula Souza[4], Eurico Arruda[4], Kirandeep Samby[6], Sujay Laskar[7], Rafael Victorio Carvalho Guido[1], Glaucius Oliva[1], Andre Schutzer Godoy[1,3*]

[1] São Carlos Institute of Physics, University of São Paulo, Av. João Dagnone, 1100 - Jardim Santa Angelina, São Carlos, 13563-120, Brazil.

[2] Calibr-Skaggs Institute for Innovative Medicine, La Jolla, CA 92037, USA.

[3] MMV Medicines for Malaria Venture, ICC, Route de Pré-Bois 20, 1215 Geneva, Switzerland.

[4] Department of Cellular and Molecular Biology and Pathogenic Bioagents, Ribeirão Preto Medical School, University of São Paulo, Ribeirão Preto 14040-900, Brazil.

[5] Departamento de Análises Clínicas, Toxicológicas e Bromatológicas, Faculdade de Ciências Farmacêuticas de Ribeirão Preto - FCFRP, Universidade de São Paulo - USP, Ribeirão Preto, 14040-903, SP, Brazil.

[6] Johnson and Johnson Pvt. Limited, India

[7] TCG Lifesciences, West Bengal, India

***Corresponding author:** andregodoy@ifsc.usp.br

Figure S1. Calculate IC_{50} of MMV1634397 and analogs against SARS-CoV-2 PL^{pro}. Error bars are SD from average of triplicates.

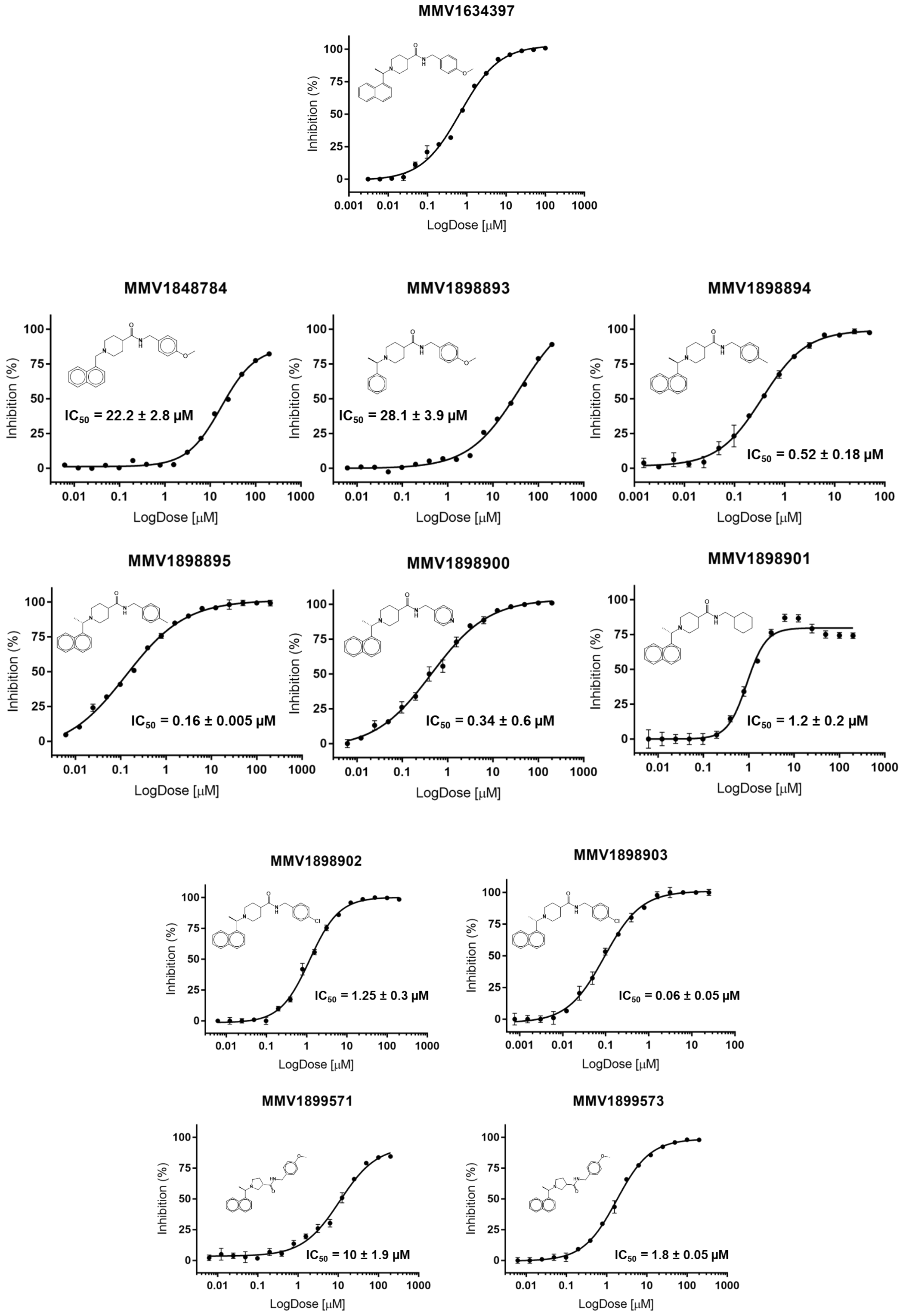

Figure S2. Curves from differential scanning fluorimetry assays of PL^{pro} in presence of compounds.

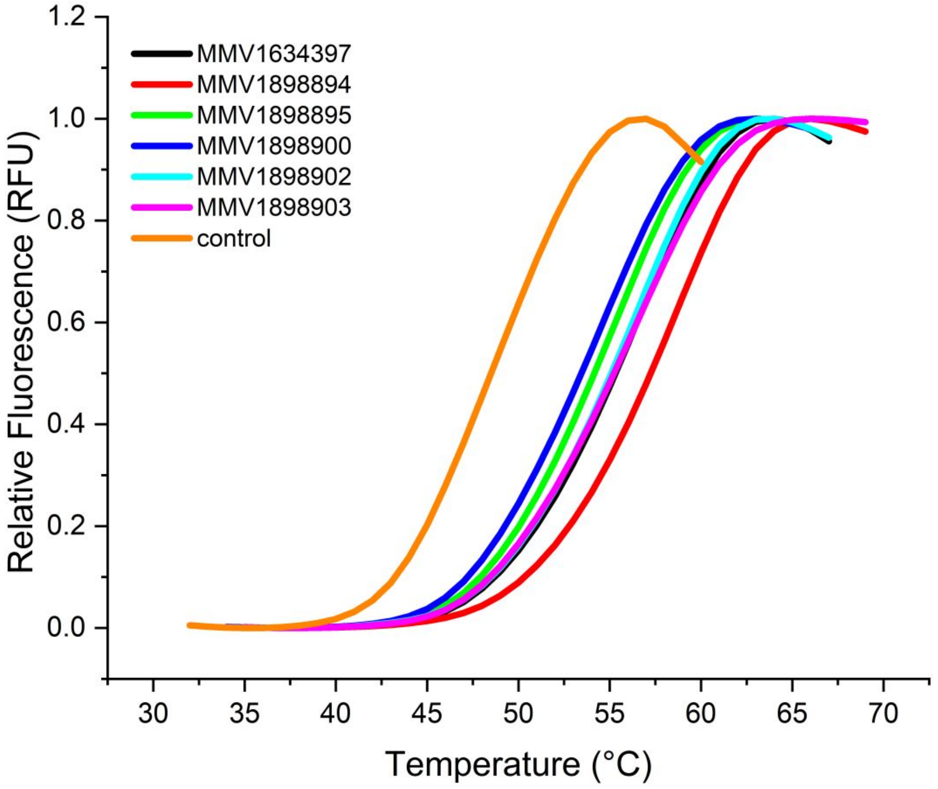

Figure S3. Structural alignment of Compound 13 (salmon), Compound 17 (pink), and the LRGG peptide (green) from the SARS-CoV PL^{pro} ubiquitin complex (PDB ID: 4MOW). (a) Binding poses shown in the same orientation as Figure 3 of the manuscript. (b) Top-down view of the active site highlighting the approximate positions of the P1–P4 substrate binding pockets, indicated in white.

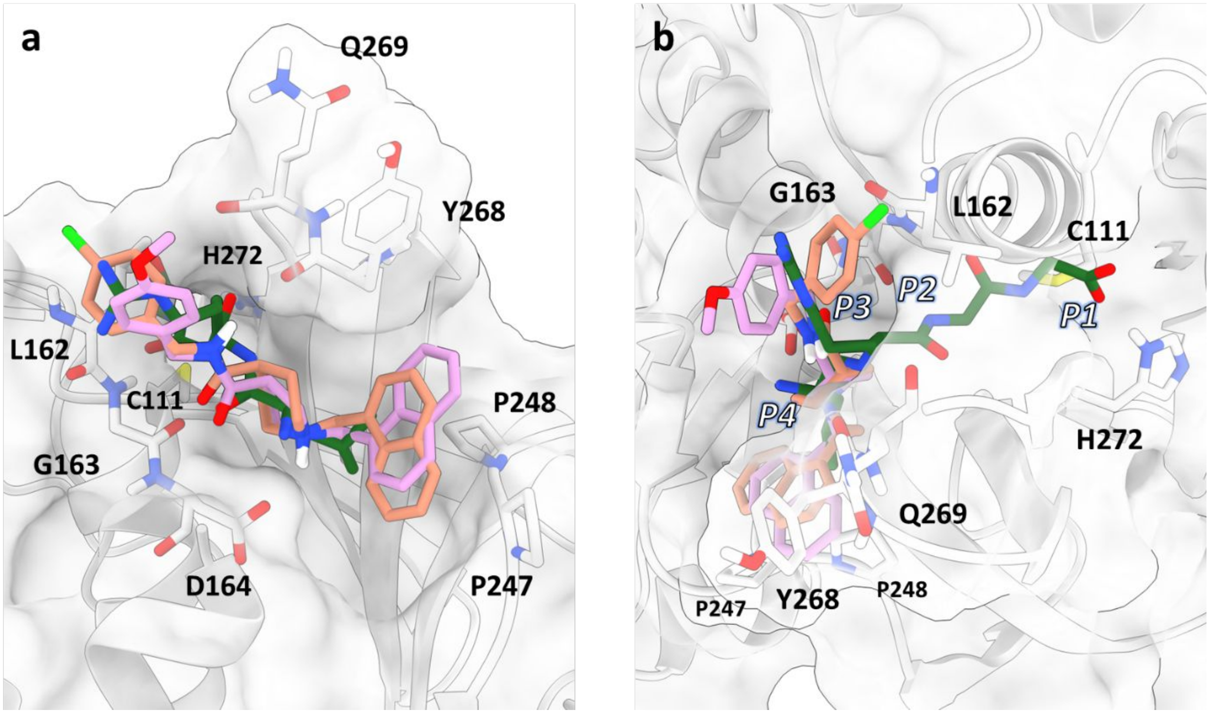

Figure S4. Dose–response curves from compounds in infection assays in ACE2-HeLa cells infected with SARS-CoV-2. The curves represent total cell count (orange), cytotoxicity in uninfected cells (red), and viable infected cells (green). Error bars indicate standard deviation (SD) from technical triplicates.

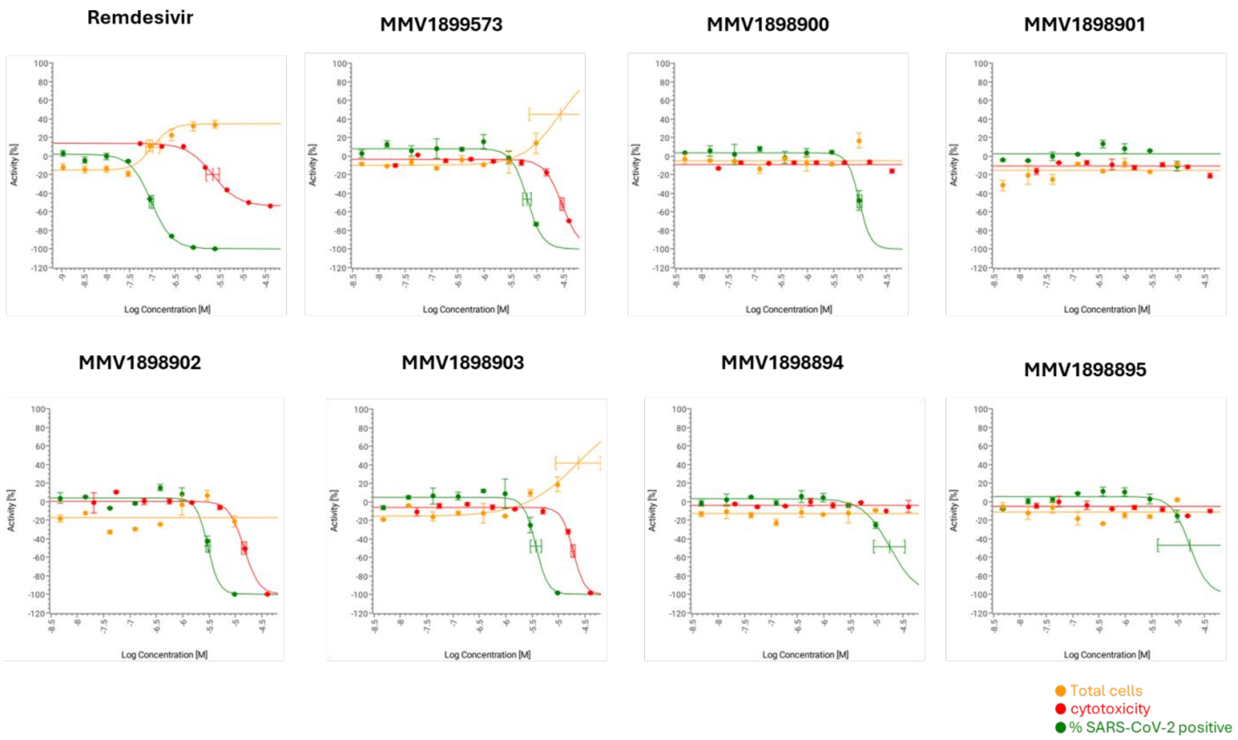

Abbreviations

Ms_2O (Mesic anhydride), **DMF** (N,N-Dimethyl formamide) **CH_3CN** (Acetonitrile), **DIPEA** (*N*,*N*-Diisopropylethylamine), **DCM** (Dichloromethane), **Na_2CO_3** (Sodium Carbonate), **$NaHCO_3$** (Sodium Bicarbonate), **Na_2SO_4** (Sodium Sulphate), **NMR** (Nuclear Magnetic Resonance), DMSO-D6 (Dimethyl sulfoxide-D6).

General synthesis procedure

The compounds of invention have been named according to the IUPAC standards used in the program Chem Bio Draw Ultra (Version 14.0). All reagents and starting materials were obtained from commercial suppliers and used without further purification. For all compounds, reaction progress was monitored by thin layer chromatography (TLC) on preloaded silica gel 60 F254 plates. Visualization was achieved with UV light and iodine vapor. Flash chromatography was carried out using prepacked Teledyne Isco Redisep™ Rf silica-gel columns as the stationary phase and analytical grade solvents as the eluent unless otherwise stated. ^{1}H nuclear magnetic resonance (NMR) spectra were recorded on a Bruker Avance II & III spectrometer, using solvents from Merck Laboratories. Chemical shifts (δ, ppm) are reported relative to the solvent peak (DMSO-d_6: 2.50 [1H]. Proton resonances are annotated as: chemical shift (δ), multiplicity (s, singlet; d, doublet; t, triplet; q, quartet; m, multiplet; br, broad), coupling constant (*J*, Hz), and number of protons.

SFC method: SFC Prep Purification was done on Waters SFC 80 instrument equipped with Waters 2489 UV/Visible Detector by using CHIRALCEL OJ-H (20.0 mm x250 mm), 5μ Column operating at 35ºC temperature, maintaining flow rate of 25 ml/min, using 75% CO2 in super critical state & 25% of 0.3% IPAmine or 0.3% DEA in Methanol/Isopropanol as Mobile phase. This isocratic mixture was run upto 10.0 minutes and also maintained the isobaric condition of 120 bar at 220 nm wavelength.

Experimental

Synthesis of (*R*)-*N*-(4-methoxybenzyl)-1-(1-phenylethyl)piperidine-4-carboxamide [Analog1_ISO-1 and of (*S*)-*N*-(4-methoxybenzyl)-1-(1-phenylethyl)piperidine-4-carboxamide [Analog1_ISO-2]

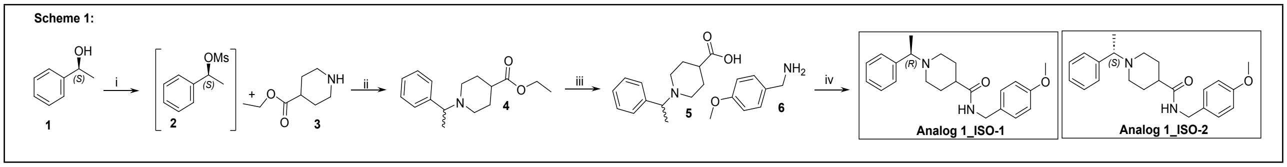

Reagent and conditions: i&ii) Ms_2O/DIPEA/MS 4A°/DCM/0°C/1h-20h (one pot); iii) (a) LiOH.H2O/THF/H2O/25°C or (b) aq.HCl/THF/60°C/16h iv) 1) HATU/DIPEA/DMF/0-25°C/16h; 2) SFC separation.

EXPERIMENTAL

Synthesis of ethyl-1-(1-phenylethyl)piperidine-4-carboxylate (**4**):

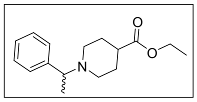

(*S*)-1-Phenylethan-1-ol (400 mg, 3.279 mmol) was dissolved in DCM (10 mL) under inert atmosphere followed by addition of freshly dried 4 Å molecular sieves. The mixture was cooled to 0 °C, then added DIPEA (1.42 mL, 9.836 mmol) and with continuous stirring Ms_2O (740 mg, 4. 262 mmol) in DCM (2 mL) was added dropwise. The solution was stirred for 1 hour at 0 °C, at which point ethyl piperidine-4-carboxylate (3.09 g, 19.672 mmol) was added. The mixture was then warmed to room temperature and allowed to stir for 20 hours. After completion of the reaction as indicated by TLC/LCMS, molecular sieves were filtered off and filtrate was diluted with DCM (30 mL), washed with 10% aqueous Na_2CO_3. The organic layer was washed with water and brine, dried over Na_2SO_4, filtered. Filtrate was evaporated to dryness to obtain crude residue which was purified by ISCO to afford ethyl-1-(1-phenylethyl)piperidine-4-carboxylate (600 mg, 70%) as pale yellow viscous liquid.

Note: Although synthesis started with pure chiral synthon (**1**), during substitution (SN2 reaction with piperidine ethyl ester) racemization observed.

^{1}H NMR (400 MHz, DMSO-D_6) δ ppm 7.35-7.18 (m, 5H), 4.04 (q, J=7.2 Hz, 2H), 3.41 (q, J=6.8 Hz, 1H), 2.86 (d, J = 11.2 Hz, 1H), 2.68 (d, J = 11.3 Hz, 1H), 2.25-2.15 (m, 1H), 1.99-1.89 (m, 2H), 1.85-1.68 (m, 2H), 1.62-1.45 (m, 2H), 1.27 (d, J = 6.7 Hz, 3H), 1.16 (t, J = 7 Hz, 3H)

LC-MS: *m/z* = 262 $[M + H]^+$

Synthesis of 1-(1-phenylethyl)piperidine-4-carboxylic acid (**5**)

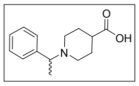

Ethyl-1-(1-phenylethyl)piperidine-4-carboxylate (250 mg, 0.842 mmol) was dissolved in 3:1:1 THF:MeOH:H_2O (15 mL) and placed in an ice-bath. LiOH.H_2O (53 mg, 1.263 mmol) was added portion-wise and the reaction mixture was stirred overnight at room temperature. On completion of the reaction as indicated by TLC, volatiles were removed under reduced pressure, acidified to pH 2 using 1N aq. HCl. Water was removed under reduced pressure to afford (*R*)-1-(1-phenylethyl)piperidine-4-carboxylic acid (**5**) (185 mg, 94%) as white solid which was carried to the next step without further purification.

Synthesis of (*R*)-*N*-(4-methoxybenzyl)-1-(1-phenylethyl)piperidine-4-carboxamide **[Analog1_ISO-1]** and of (*S*)-*N*-(4-methoxybenzyl)-1-(1-phenylethyl)piperidine-4-carboxamide **[Analog1_ISO-2]**

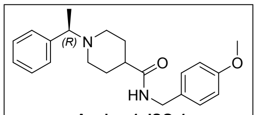

Analog 1_ISO-1

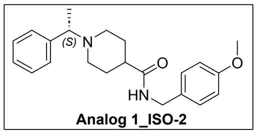

Analog 1_ISO-2

To a stirred solution of 1-(1-phenylethyl)piperidine-4-carboxylic acid (**5**) (300 mg, 1.286 mmol) in DMF (10 mL) under inert atmosphere were added HATU (932 mg, 1.929 mmol), DIPEA (1 mL) followed by addition of (4-methoxyphenyl)methanamine (**6**) (194 mg, 1.414 mmol) in DMF (1 mL) at 0 °C and the reaction was stirred at 25 °C for 12 hours. After completion of the reaction as indicated by TLC/LCMS, the mixture was added with water and extracted with ethyl acetate. The combined organic layer was washed with water, brine and dried over anhydros sodium sulphate. Filtered and the filtrate was evaporated under reduced pressure to afford crude residue which was purified by ISCO to afford (*R*)-N-(4-methoxybenzyl)-1-(1-phenylethyl)piperidine-4-carboxamide (400 mg, 88%) as white solid with enantiomeric excess (ee)>62%.

Note: Enantiomers were separated by SFC purification; Stereochemical assigment was done arbitrarily.

<u>Analog1 ISO-1;</u>

^{1}H NMR (400 MHz, DMSO-D_6) *δ* ppm 8.12 (t, J = 5.8 Hz, 1H), 7.35-7.18 (m, 5H), 7.12 (d, J = 8.5 Hz, 2H), 6.85 (d, J = 8.5 Hz, 2H), 4.15 (d, J = 5.8 Hz, 2H), 3.71 (s, 3H), 3.43-3.38 (m, 1H), 2.95 (d, J = 11.2 Hz, 1H), 2.74 (d, J = 11.48 Hz, 1H), 2.10-2.00 (m, 1H), 1.95-1.70 (m, 2H), 1.65-1.50 (m, 4H), 1.25 (d, J = 6 Hz, 3H).

LC-MS: *m/z* = 353 $[M + H]^+$; **HPLC**: 99.52%; **ee**: >99%

Analog1 ISO-2;

^{1}**H NMR** (400 MHz, DMSO-D_6) δ ppm 8.12 (t, J = 5.6 Hz, 1H), 7.40-7.15 (m, 5H), 7.12 (d, J = 8.5 Hz, 2H), 6.85 (d, J = 8.6 Hz, 2H), 4.15 (d, J = 5.8 Hz, 2H), 3.71 (s, 3H), 3.45-3.35 (m, 1H), 2.95 (d, J = 10.8 Hz, 1H), 2.74 (d, J = 11.6 Hz, 1H), 2.10-2.00 (m, 1H), 1.95-1.70 (m, 2H), 1.65-1.50 (m, 4H), 1.25 (d, J = 6 Hz, 3H).

LC-MS: *m/z* = 353 $[M + H]^+$; **HPLC**: 97.50%; **ee**: >99%

Synthesis of *N*-(4-methoxybenzyl)-1-(naphthalen-1-ylmethyl)piperidine-4-carboxamide [Analog 2]

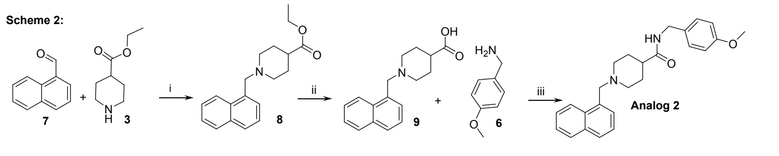

Reagent and conditions: i) 4A° MS/$NaCNBH_4$/THF/0°C/1h-20h; ii) $LiOH.H_2O$/THF/H_2O/25°C; iii) HATU DIPEA DMF/0-25°C/16h

Synthesis of ethyl 1-(naphthalen-1-ylmethyl)piperidine-4-carboxylate (8):

To a stirred solution of 1-naphthaldehyde (**7**) (500 mg, 3.205 mmol), and ethyl piperidine-4-carboxylate (**3**) (503 mg, 3.205 mmol) in THF (10 mL), was added molecular sieves followed by addition of $NaCNBH_3$ (302 mg, 4.808 mmol) and stirred at 25°C for overnight. After completion of the reaction as indicated by TLC/LCMS, reaction crude was filtered and purified by ISCO to afford

ethyl 1-(naphthalen-1-ylmethyl)piperidine-4-carboxylate (**8**) (250 mg, 26%) as a white solid with close impurities which was carried to the next step without further purification.

LC-MS: *m/z* = 298 [M + H]$^+$

Synthesis of 1-(naphthalen-1-ylmethyl)piperidine-4-carboxylic acid (9)

To a stirred solution of ethyl 1-(naphthalen-1-ylmethyl)piperidine-4-carboxylate (**8**) (250 mg) in THF (4 mL) and H_2O (2 mL) were added with $LiOH.H_2O$ (178.95 mg, 4.27 mmol) at 25° C and mixture was continued to stir at 25°C for 6 hours. After completion of reaction as judged by LCMS/TLC, the mixture was added with ice water and acidified with 1 N HCl. A white coloured precipitate was formed. The solid was filtered and washed with water and azeotroped with toluene to afford 1-(naphthalen-1-ylmethyl)piperidine-4-carboxylic acid (**9**) (200 mg, 88%) as off-white solid, which was carried to the next step without further purification.

Synthesis of N-(4-methoxybenzyl)-1-(naphthalen-1-ylmethyl)piperidine-4-carboxamide [Analog 2]

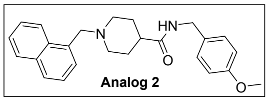

Synthesis of *N*-(4-methoxybenzyl)-1-(naphthalen-1-ylmethyl)piperidine-4-carboxamide has been achieved by following similar procedure described for Analog 1 using 1-(naphthalen-1-ylmethyl)piperidine-4-carboxylic acid (**9**) (100 mg, 0.371 mmol) and p-methoxybenzylamine (**6**) (61 mg, 0.44 mmol) to afford *N*-(4-methoxybenzyl)-1-(naphthalen-1-ylmethyl)piperidine-4-carboxamide (**Analog 2**) (80 mg, 55%) as off white solid.

Analog 2

^{1}H NMR (400 MHz, DMSO-D_6) δ ppm 8.27 (d, J = 7.8 Hz, 1H), 8.18 (bs, 1H), 7.90 (d, J = 7.8 Hz, 1H), 7.83 (bs, 1H), 7.58-7.41 (m, 4H), 7.12 (d, J = 8.4 Hz, 2H), 6.85 (d, J = 8.5 Hz, 2H), 4.15 (d, J = 5.8 Hz, 2H), 3.83 (s, 2H), 3.70 (s, 3H), 2.88 (d, J = 11.6 Hz, 2H), 2.24-2.10 (m, 1H), 1.99 (t, J = 10.6 Hz, 2H), 1.70-1.50 (m, 4H).

LC-MS: *m/z* = 389.33 [M + H]$^+$; **HPLC**: 99.94%

Synthesis of *N*-(cyclohexylmethyl)-1-(1-(naphthalen-1-yl)ethyl)piperidine-4-carboxamide **[Analog3]**

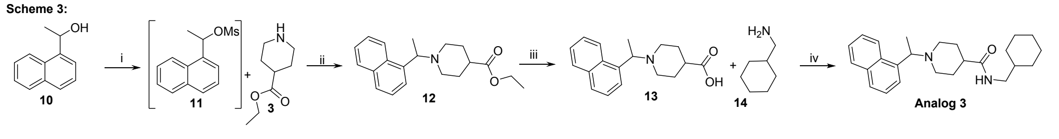

Reagent and conditions: i & ii) Ms_2O/DIPEA/MS4A°/DCM/0°C/1h-20h iii) LiOH.H2O/THF/H2O/25°C iv) HATU/DIPEA/DMF/0-25°C/16h

Synthesis of ethyl-(1-(naphthalen-1-yl)ethyl)piperidine-4-carboxylate (**12**):

1-(Naphthalen-1-yl)ethan-1-ol (**10**) (500 mg, 2.903 mmol) was dissolved in DCM (10 mL) and added 4 Å molecular sieves. Cooled to 0°C followed by addition of DIPEA (1.5 mL, 8.7 mmol) was added dropwise. With continuous stirring at 0 °C, Ms_2O (657 mg, 3.774 mmol) in DCM (2 mL) was added dropwise. The mixture was stirred for 1 hour at 0 °C, at which point ethyl piperidine-4-carboxylate (**3**) (2.7g, 17.42 mmol) was added and continued the stirring for 20 hours at room temperature. After completion of the reaction as indicated by TLC/LCMS, molecular sieves were

filtered off and filtrate was diluted with DCM (30 mL), washed with 10% aqueous Na_2CO_3. The organic layer was washed with water and brine, dried over Na_2SO_4, filtered. Filtrate was evaporated to dryness to obtain crude residue which was purified by ISCO to afford ethyl-1-(1-(naphthalen-1-yl)ethyl)piperidine-4-carboxylate (**12**) (700 mg, 77%) as pale yellow viscous liquid.

^{1}H NMR (400 MHz, DMSO-D_6) δ ppm 8.42 (d, J = 7.5 Hz, 1H), 7.70 (d, J = 6.4 Hz, 1H), 7.79 (d, J = 7.8 Hz, 1H), 7.60-7.40 (m, 4H), 4.20-4.10 (m, 1H), 4.03 (q, J =7.2 Hz, 2H), 2.96 (d, J = 11 Hz, 1H), 2.72 (d, J = 10.9 Hz, 1H), 2.25 (t, J = 11 Hz, 1H), 2.12-2.02 (m, 2H), 1.79 (d, J = 12.3 Hz, 1H), 1.70 (d, J = 11.6 Hz, 1H), 1.55-1.45 (m, 2H), 1.38 (d, J = 6.5 Hz, 3H)

LC-MS: *m/z* = 312 $[M + H]^+$;

<u>Synthesis of 1-(1-(naphthalen-1-yl)ethyl)piperidine-4-carboxylic acid (**13**):</u>

Ethyl-1-(1-(naphthalen-1-yl)ethyl)piperidine-4-carboxylate (**12**) (700 mg, 2.24 mmol) was dissolved in 3:1:1 THF:MeOH:H2O (15 mL) and placed in an ice-bath $LiOH.H_2O$ (138 mg, 3.3 mmol) was added portion-wise and the reaction mixture was stirred overnight at room temperature. On completion of the reaction as indicated by TLC/LCMS, volatiles were evaporated to dryness and re-dissolved in water (4 mL) and the pH was adjusted to 2 (with 1N aq. HCl) at 0°C. White solid precipitated which was filtered and washed with water and dried to afford 1-(1-(naphthalen-1-yl)ethyl)piperidine-4-carboxylic acid (**13**) (600 mg, 94%) as a white solid.

LC-MS: *m/z* = 284 $[M + H]^+$;

<u>Synthesis of *N*-(cyclohexylmethyl)-1-(1-(naphthalen-1-yl)ethyl)piperidine-4-carboxamide [**Analog3**]</u>

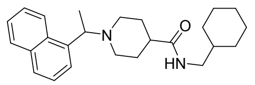

Analog 3

Synthesis of *N*-(cyclohexylmethyl)-1-(1-(naphthalen-1-yl)ethyl)piperidine-4-carboxamide (**Analog 3**) has been achieved by following similar procedure described for Analog 1 using 1-(1-(naphthalen-1-yl)ethyl)piperidine-4-carboxylic acid (**5**) (350 mg, 1.23 mmol) and cyclohexylmethanamine (**6**) (0.193 mL, 1.48 mmol) to afford *N*-(cyclohexylmethyl)-1-(1-(naphthalen-1-yl)ethyl)piperidine-4-carboxamide (Analog) (150 mg, 32%) as off white solid.

^{1}H NMR (400 MHz, DMSO-D$_6$) δ ppm 8.45 (d, J = 7.9 Hz, 1H), 7.90 (d, J = 7.1 Hz, 1H), 7.79 (d, J = 8Hz, 1H), 7.63 (t, J = 5.6 Hz, 1H), 7.55-7.43 (m, 4H), 4.12 (q, J = 6.6 Hz, 1H), 3.05 (d, J = 11.2 Hz, 1H), 2.84 (t, J = 6.3 Hz, 2H), 2.76 (d, J = 11.1 Hz, 1H), 2.15-1.90 (m, 3H), 1.70-1.45 (m, 8H), 1.39 (d, J = 6.6 Hz, 3H), 1.37-1.31 (m, 1H), 1.20-1.05 (m, 3H), 0.90-0.75 (m, 2H)

LC-MS: *m/z* = 379 [M + H]$^+$;

<u>Synthesis of **(*R*)-*N*-(4-chlorobenzyl)-1-(1-(naphthalen-1-yl)ethyl)piperidine-4-carboxamide [Analog4_ISO-1] & (*S*)-*N*-(4-chlorobenzyl)-1-(1-(naphthalen-1-yl)ethyl)piperidine-4-carboxamide [Analog4_ISO-2]**</u>

Scheme 4:

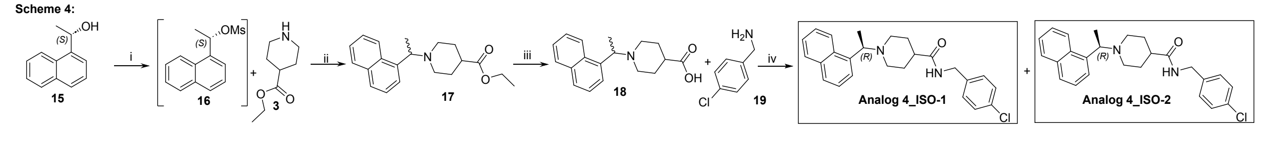

Reagent and conditions: i&ii) Ms$_2$O/DIPEA/MS4A°/DCM/0°C/1h-20h iii) LiOH.H2O/THF/H2O 25°C iv) 1) HATU/DIPEA/DMF/0-25°C/16h; 2) SFC separation.

Synthesis of ethyl-1-(1-(naphthalen-1-yl)ethyl)piperidine-4-carboxylate (**17**):

Synthesis of ethyl-1-(1-(naphthalen-1-yl)ethyl)piperidine-4-carboxylate (**17**) was achieved by following the similar procedure described for racemic version (**Int-12**, **Scheme-3**) using (*S*)-1-(naphthalen-1-yl)ethan-1-ol (**15**) (500 mg, 2.903 mmol), DIPEA (1.5 mL, 8.7 mmol), Ms_2O (657 mg, 3.774 mmol) and ethyl piperidine-4-carboxylate (**3**) (2.7g, 17.42 mmol) to afford ethyl-1-(1-(naphthalen-1-yl)ethyl)piperidine-4-carboxylate (**17**) (700 mg, 77%) to afford a pale yellow liquid which was carried to the next step without further analysis.

Note: Although synthesis started with pure chiral synthon (**15**), during substitution (SN2 reaction with piperidine ethyl ester) racemization observed.

Synthesis of 1-(1-(naphthalen-1-yl)ethyl)piperidine-4-carboxylic acid (**18**):

Synthesis of 1-(1-(naphthalen-1-yl)ethyl)piperidine-4-carboxylic acid (**18**) was achieved by following the similar procedure described for racemic version (**Int-13, Scheme 3**) using ethyl-1-(1-(naphthalen-1-yl)ethyl)piperidine-4-carboxylate (**17**) (700 mg, 2.24 mmol) and $LiOH.H_2O$ (xx mg, mmol) to afford 1-(1-(naphthalen-1-yl)ethyl)piperidine-4-carboxylic acid (**18**) as white solid with minor impurities which was carried to the next step without further purification.

LC-MS: *m/z* = 284 $[M + H]^+$;

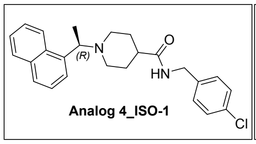

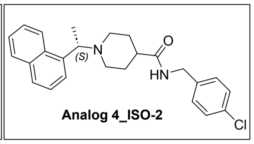

Synthesis of *N*-(4-chlorobenzyl)-1-(1-(naphthalen-1-yl)ethyl)piperidine-4-carboxamide (**Analog 4**):

Synthesis of *N*-(4-chlorobenzyl)-1-(1-(naphthalen-1-yl)ethyl)piperidine-4-carboxamide (**Analog 4**) has been synthesized following similar procedure described for Analog 1 using 1-(1-(naphthalen-1-yl)ethyl)piperidine-4-carboxylic acid (**18**) (350 mg, 1.235 mmol), HATU (705 mg, 1.85 mmol), DIPEA (0.65 mL, 3.7 mmol) and p-chlorobenzylamine (**19**) (0.165 mL, 1.235 mmol) to afford racemic *N*-(4-chlorobenzyl)-1-(1-(naphthalen-1-yl)ethyl)piperidine-4-carboxamide (**Analog 4**) (150 mg, 29%) as white solid which was further subjected to SFC separation to obtain respective enantiomers (*R*)-*N*-(4-chlorobenzyl)-1-(1-(naphthalen-1-yl)ethyl)piperidine-4-carboxamide **Analog4_ISO-1 and** (*S*)-*N*-(4-chlorobenzyl)-1-(1-(naphthalen-1-yl)ethyl)piperidine-4-carboxamide **Analog4_ISO-2.**

Note: Enantiomers were separated by SFC purification; Stereochemical assigment was done arbitrarily.

Analog4_ISO-1

^{1}**H NMR** (400 MHz, DMSO-D_6) δ ppm 8.45 (d, J = 7.8 Hz, 1H), 8.27 (t, J = 5.8 Hz, 1H), 7.90 (d, J = 6.6 Hz, 1H), 7.79 (d, J = 8 Hz, 1H), 7.55-7.40 (m, 4H), 7.34 (d, J = 8.3 Hz, 2H), 7.21 (d, J = 8.3 Hz, 2H), 4.20 (d, J = 5.8 Hz, 2H), 4.14 (q, J = 6.4 Hz, 1H), 3.06 (d, J = 10.5 Hz, 1H), 2.78 (d, J = 11 Hz, 1H), 2.18-2.14 (m, 1H), 2.05-1.95 (m, 2H), 1.75-1.65 (m, 1H), 1.62-1.45 (m, 3H), 1.39 (d, J = 6.6 Hz, 3H).

LC-MS: *m/z* = 407 $[M + H]^+$; **HPLC**: 99.90%; **ee**>99%

Analog4_ISO-2

¹H NMR (400 MHz, DMSO-D_6) δ ppm 8.45 (d, J = 7.7 Hz, 1H), 8.27 (t, J = 5.4 Hz, 1H), 7.90 (d, J = 6.6 Hz, 1H), 7.79 (d, J = 8 Hz, 1H), 7.55-7.40 (m, 4H), 7.34 (d, J = 8.12 Hz, 2H), 7.21 (d, J = 8 Hz, 2H), 4.21 (d, J = 5.7 Hz, 2H), 4.13 (q, J = 6.4 Hz, 1H), 3.06 (d, J = 10.6 Hz, 1H), 2.78 (d, J = 10.9 Hz, 1H), 2.18-2.14 (m, 1H), 2.05-1.95 (m, 2H), 1.75-1.65 (m, 1H), 1.60-1.45 (m, 3H), 1.39 (d, J = 6.6 Hz, 3H).

LC-MS: *m/z* = 407 $[M + H]^+$; **HPLC**: 99.85%; **ee**>98%

Synthesis of (*R*)-*N*-(4-methylbenzyl)-1-(1-(naphthalen-1-yl)ethyl)piperidine-4-carboxamide [Analog5_ISO-1] and (*S*)-N-(4-methylbenzyl)-1-(1-(naphthalen-1-yl)ethyl)pipe-ridine-4-carboxamide [Analog5_ISO-2]

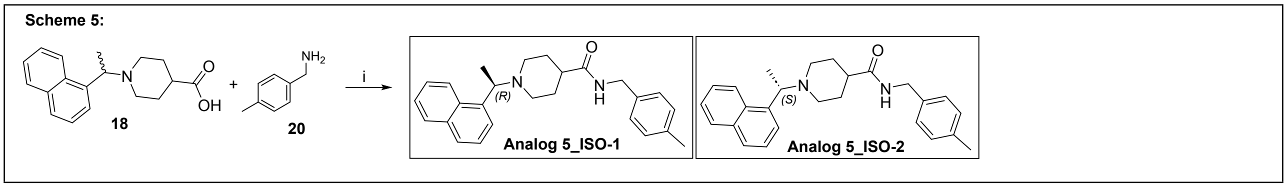

Reagent and conditions: A) i) HATU/DIPEA/DMF/0-25°C/16h; ii) SFC separation.

Synthesis of (*R*)-*N*-(4-methylbenzyl)-1-(1-(naphthalen-1-yl)ethyl)piperidine-4-carboxamide **[Analog5_ISO-1]** and (*S*)-N-(4-methylbenzyl)-1-(1-(naphthalen-1-yl)ethyl)piperidine-4-carboxamide **[Analog5_ISO-2]**

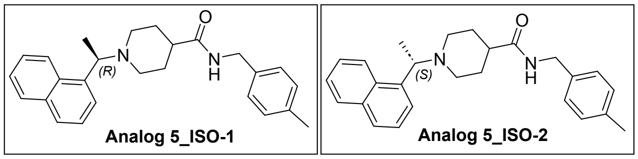

Synthesis of (*R*)-*N*-(4-methylbenzyl)-1-(1-(naphthalen-1-yl)ethyl)piperidine-4-carboxamide **[Analog5_ISO-1]** and (*S*)-N-(4-methylbenzyl)-1-(1-(naphthalen-1-yl)ethyl)piperidine-4-carboxamide **[Analog5_ISO-2]** was achieved by following the similar procedure described for Analog 1 (Scheme 1) utilizing 1-(1-(naphthalen-1-yl)ethyl)piperidine-4-carboxylic acid (**18**) (125 mg, 0.433 mmol) and 4-methylbenzylamine (**20**) (58 mg, 0.477 mmol) to afford racemic **Analog-5** (100 mg, yield: 60%) which was further subjected to SFC separation of enanantiomers to obtain respective enanitomers (*R*)-*N*-(4-methylbenzyl)-1-(1-(naphthalen-1-yl)ethyl)piperidine-4-carboxamide **[Analog5_ISO-1]** (32 mg, **ee**>99%) and (*S*)-N-(4-methylbenzyl)-1-(1-(naphthalen-1-yl)ethyl)piperidine-4-carboxamide **[Analog5_ISO-2]** (77 mg, **ee**>99%).

Note: Enantiomers were separated by SFC purification; Stereochemical assigment was done arbitrarily.

Analog5_ISO-1

^{1}H NMR (400 MHz, DMSO-D$_6$) δ ppm 8.45 (d, J = 7.9 Hz, 1H), 8.17 (t, J = 5.8 Hz, 1H), 7.90 (d, J = 7.2 Hz, 1H), 7.79 (d, J = 8 Hz, 1H), 7.55-7.40 (m, 4H), 7.08 (s, 4H), 4.18-4.13 (m, 3H), 3.06 (d, J = 10.8 Hz, 1H), 2.78 (d, J = 11.3 Hz, 1H), 2.25 (s, 3H), 2.16-1.92 (m, 3H), 1.70-1.65 (m, 1H), 1.62-1.50 (m, 3H), 1.39 (d, J = 6.6 Hz, 3H).

LC-MS: *m/z* = 387.41 $[M + H]^+$; **HPLC**: 99.87%; **ee**>99%

Analog5_ISO-2

^{1}H NMR (400 MHz, DMSO-D$_6$) δ ppm 8.45 (d, J = 7.9 Hz, 1H), 8.16 (t, J = 5.9 Hz, 1H), 7.90 (d, J = 7.2 Hz, 1H), 7.79 (d, J = 8 Hz, 1H), 7.56-7.42 (m, 4H), 7.08 (s, 4H), 4.18 -4.12 (m, 3H), 3.06 (d, J = 10.8 Hz, 1H), 2.78 (d, J = 11.3 Hz, 1H), 2.25 (s, 3H), 2.16-1.92 (m, 3H), 1.75-1.65 (m, 1H), 1.62-1.45 (m, 3H), 1.39 (d, J = 6.6 Hz, 3H).

LC-MS: *m/z* = 387.41 $[M + H]^+$; **HPLC**: 98.84%; **ee**>99%

Synthesis of 1-(1-(naphthalen-1-yl)ethyl)-*N*-(pyridin-4-ylmethyl)piperidine-4-carboxamide [Analog6]

Scheme 6:

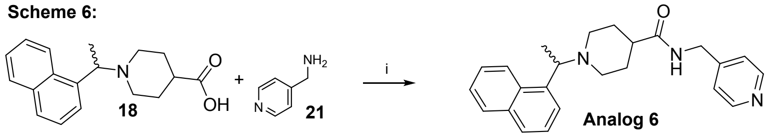

Reagent and conditions: i) HATU/DIPEA/DMF/0-25°C/16h;

Synthesis of 1-(1-(naphthalen-1-yl)ethyl)-*N*-(pyridin-4-ylmethyl)piperidine-4-carboxamide (**Analog 6**) has been achieved by following the similar procedure described for Analog 1 (**Scheme 1**) utilizing 1-(1-(naphthalen-1-yl)ethyl)piperidine-4-carboxylic acid (**18**) (350 mg, 1.23 mmol) and pyridin-4-ylmethanamine (**21**) (0.193 mL, 1.48 mmol) to afford 1-(1-(naphthalen-1-yl)ethyl)-*N*-(pyridin-4-ylmethyl)piperidine-4-carboxamide [**Analog 6**] compound (150 mg, 32%) as off white solid .

Analog 6

^{1}H NMR (400 MHz, DMSO-D_6) δ ppm 8.55 (m, 3H), 8.34 (t, J = 5.8 Hz, 1H), 7.90 (d, J = 7.2 Hz, 1H), 7.79 (d, J = 8Hz, 1H), 7.60-7.45 (m 4H), 7.17 (d, J = 5.6 Hz, 2H), 4.25 (d, J = 5.9 Hz, 2H), 4.15 (q, J = 6.6 Hz, 1H), 3.07 (d, J = 10.9 Hz, 1H), 2.79 (d, J = 11.2 Hz, 1H), 2.25-2.15 (m, 1H), 2.05-1.95 (m 2H), 1.80-1.70 (m, 1H), 1.70-1.50 (m, 3H), 1.39 (d, J = 6.6 Hz, 3H).

LC-MS: *m/z* = 374 $[M + H]^+$; **HPLC**: 99.67%;

Synthesis of 1-(sec-butyl)-*N*-(4-methoxybenzyl)piperidine-4-carboxamide [Analog 7]

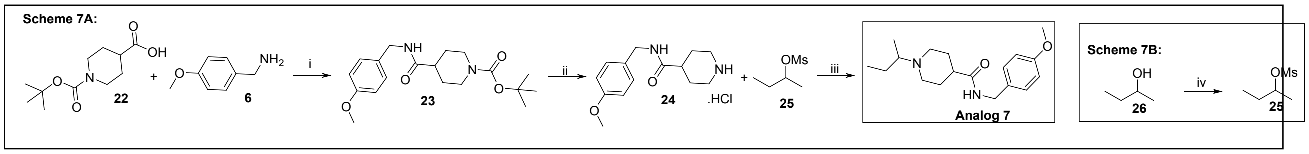

Reagent and conditions: i) EDC, HOBt, DIPEA, DMF, 0-25°C,16h; ii) HCl/ether, 0-25°C, 2h; iii) K_2CO_3, DMF/CAN, reflux; iv) MsCl, TEA, DCM,

Synthesis of *tert*-butyl 4-((4-methoxybenzyl)carbamoyl)piperidine-1-carboxylate (**23**):

To a stirred solution of 1-(*tert*-butoxycarbonyl)piperidine-4-carboxylic acid (**22**) (500 mg, 2.18 mmol) in DMF (3 mL) at 0°C under inert atmosphere were added DIPEA (1.13 mL, 6.54 mmol) drop-wise followed by the addition of EDC.HCl (627 mg, 3.27 mmol) and HOBt (442 mg, 3.27 mmol). The reaction mixture was allowed to stir for 20 minutes at room temperature followed by the addition of p-methoxy benzylamine (0.28 mL, 2.18 mmol). Then the mixture was stirred for overnight at room temperature. After completion of the reaction as indicated by TLC/LCMS, the mixture was added with water and extracted with ethyl acetate. The combined organic layer was washed with water, brine and dried over anhydrous sodium sulphate. Filtered and the filtrate was evaporated under reduced pressure to afford crude residue which was purified by ISCO column to afford *tert*-butyl 4-((4-methoxybenzyl)carbamoyl)piperidine-1-carboxylate (**23**) (200 mg, 26%) as white solid.

^{1}H NMR (400 MHz, DMSO-D_6) δ ppm 8.24 (t, J = 5.6 Hz, 1H), 7.14 (d, J = 8.5 Hz, 2H), 6.86 (d, J = 8.6 Hz, 2H), 4.17 (d, J = 5.8 Hz, 2H), 3.92 (d, J = 13.9 Hz, 2H), 3.71 (s, 3H), 2.80-2.65 (bs, 2H), 2.34-2.29 (m, 1H), 1.65 (d, J = 11 Hz, 2H), 1.50-1.30 (m, 11H)

LC-MS: *m/z* = 349 $[M + H]^+$

Synthesis of N-(4-methoxybenzyl)piperidine-4-carboxamide hydrochloride (**24**):

HCl in ether (10 mL) was added to *tert*-butyl 4-((4-methoxybenzyl)carbamoyl)piperidine-1-carboxylate (**23**) (300 mg, 0.862 mmol) at 0°C and stirred for 6 hours at room temperature. The reaction was monitored by LCMS. On completion the volatiles were evaporated under reduced pressure to afford *N*-(4-methoxybenzyl)piperidine-4-carboxamide hydrochloride (**24**) (200 mg, 93%) as white solid which was carried to the next step without further purification.

LC-MS: *m/z* = 249 $[M + H]^+$

Synthesis of sec-butyl methanesulfonate (**25**):

sec-Butanol (1.7g, 22.97 mmol) was dissolved in DCM (29 mL) under inert atmosphere and cooled to 0 °C. TEA (4.7 mL, 34.45 mmol) was added drop-wise, followed by the addition of methanesulfonyl chloride (1.7 mL, 22.97 mmol) drop-wise. On completion, as indicated by TLC, diluted with DCM (30 mL) added with water, extracted with DCM, washed with sat. aq. Na_2CO_3, water and brine. Dried over sodium sulphate, filtered and evaporated to dryness under reduced pressure to afford sec-butyl methanesulfonate (**25**) (1.8g, 51%) as pale yellow liquid.

^{1}H NMR (400 MHz, DMSO-D_6) δ ppm 4.76-4.68 (m, 1H), 2.96 (s, 3H), 1.80-1.60 (m, 2H), 1.39 (d, J = 6.2 Hz, 3H), 0.96 (t, J = 7.4 Hz, 3H).

Synthesis of 1-(sec-butyl)-*N*-(4-methoxybenzyl)piperidine-4-carboxamide **[Analog 7]**

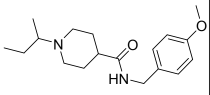

Analog 7

N-(4-methoxybenzyl)piperidine-4-carboxamide hydrochloride (**24**) (100 mg, 0.403 mmol) and sec-butyl methanesulfonate (**25**) (61 mg, 0.403 mmol) were dissolved in DMF/ACN (2 mL, 1:1) under inert atmosphere followed by addition of K_2CO_3 (55 mg, 0.403 mmol). The mixture was heated at 95 °C overnight. After completion of the reaction as indicated by TLC/LCMS, water was added and extracted with EtOAc. The combined organic layer was washed with brine and dried over Na2SO4, filtered and filtrate was evaporated under reduced pressure to obtain crude residue which was purified by ISCO column to afford 1-(sec-butyl)-*N*-(4-methoxybenzyl)piperidine-4-carboxamide (23 mg, 18%) as off-white sticky solid.

^{1}H NMR (400 MHz, DMSO-D$_6$) δ ppm 8.15 (t, J = 5.7 Hz, 1H), 7.14 (d, J = 8.5 Hz, 2H), 6.86 (d, J = 8.5 Hz, 2H), 4.16 (d, J = 5.8 Hz, 2H), 3.71 (s, 3H), 2.72-2.65 (m, 2H), 2.45-2.35 (m, 1H), 2.22 (t, J = 11.3 Hz, 1H), 2.10-2.00 (m, 2H), 1.70-1.40 (m, 5H), 1.25-1.15 (m, 1H), 0.88-0.80 (m, 6H).

LC-MS: *m/z* = 305 [M + H]$^+$; **HPLC**: 97.68%;

Synthesis of *N*-(4-methoxybenzyl)-1-((*R*)-1-(naphthalen-1-yl)ethyl)pyrrolidine-3-carboxamide and its 3 other diastereomers [(Analog8_ISO-1; Analog8_ISO-2; Analog8_ISO-3; and Analog8_ISO-4]

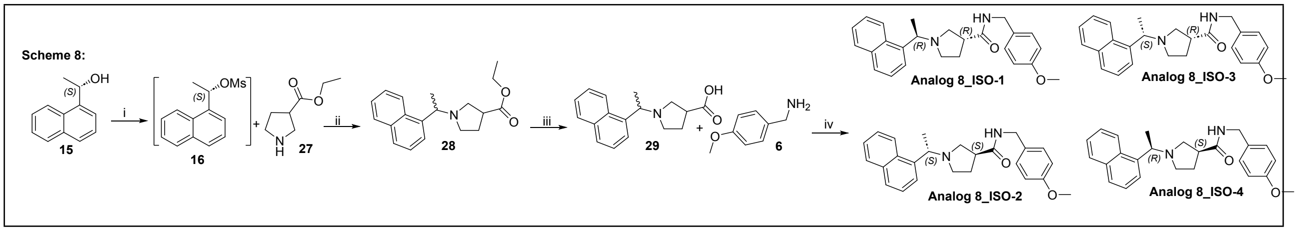

Reagent and conditions: : i & ii) Ms_2O/DIPEA/MS4A°/DCM/0°C/1h-20h iii) LiOH.H2O/THF/H2O/25°C iv) 1) HATU/DIPEA/DMF/0-25°C/16h; 2) SFC separation.

Synthesis of ethyl 1-((R)-1-(naphthalen-1-yl)ethyl)pyrrolidine-3-carboxylate (**28**)

Synthesis of ethyl 1-(naphthalen-1-yl)ethyl)pyrrolidine-3-carboxylate (**28**) was achieved by following the similar procedure described for compound 4 (Scheme 1) using (*S*)-1-(naphthalen-1-yl)ethan-1-ol (**15**) (500 mg, 2.903 mmol), DIPEA (1.5 mL, 8.7 mmol), Ms_2O (657 mg, 3.774 mmol) and ethyl pyrrolidine-3-carboxylate (**27**) (1.04g, 5.8 mmol) to afford ethyl 1-(1-(naphthalen-1-yl)ethyl)pyrolidine-3-carboxylate (**28**) (450 mg, 52%) as a pale yellow liquid.

Note: Although synthesis started with pure chiral synthon (**15**), during substitution reaction racemization observed.

^{1}H NMR (400 MHz, DMSO-D_6) δ ppm 8.44 (bs, 1H), 7.95-7.90 (m, 1H), 7.79 (d, J = 7.4 Hz, 1H), 7.64-7.56 (m, 1H), 7.55-7.42 (m, 3H), 4.15-3.98 (m, 2H), 3.00-2.90 (m, 1H), 2.80-2.60 (m, 2H), 2.05-1.90 (m, 2H), 1.41 (d, J = 4.5 Hz, 3H), 1.11 (t, J = 7.2 Hz, 3H).

LC-MS: *m/z* = 305 $[M + H]^+$

Synthesis of 1-(1-(naphthalen-1-yl)ethyl)pyrrolidine-3-carboxylic acid (**29**)

Synthesis of 1-(1-(naphthalen-1-yl)ethyl)pyrrolidine-3-carboxylic acid (**29**) was achieved by following the similar procedure described in scheme 1 using ethyl 1-(1-(naphthalen-1-yl)ethyl)pyrolidine-3-carboxylate (**28**) and LiOH.H2O (93 mg, 2.222 mmol) to afford 1-(1-(naphthalen-1-yl)ethyl)pyrrolidine-3-carboxylic acid (**29**) (390 mg, 98%) as off-white solid which was carried to the next step without further purification.

LC-MS: *m/z* = 270 [M + H]$^+$

<u>Synthesis of N-(4-methoxybenzyl)-1-((R)-1-(naphthalen-1-yl)ethyl)pyrrolidine-3-carboxamide and its 3 other diastereomers</u>

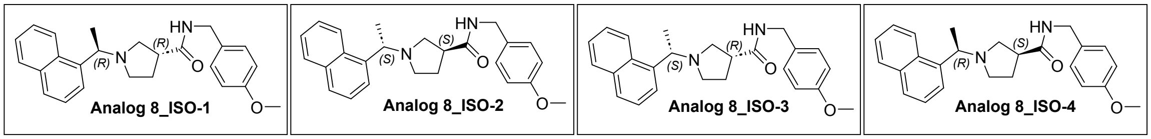

Synthesis has been achieved following the similar procedure described for Analog3 (**Scheme 3**) utilizing 1-(1-(naphthalen-1-yl)ethyl)pyrrolidine-3-carboxylic acid (**29**) (398 mg, 1.48 mmol) and p-methoxy benzylamine (**6**) (0.2 mL, 1.62 mmol), HATU (843 mg, 2.21 mmol) and DIPEA (0.78 mL, 4.434 mmol) to afford racemic *N*-(4-methoxybenzyl)-1-(1-(naphthalen-1-yl)ethyl)pyrrolidine-3-carboxamide (300 mg, 52%) as a white solid. This racemic mixture was further subjected to chiral SFC purification to obtain four diastereomers [44 mg, **Analog8_ISO-1**]**,** [22 mg, **Analog8_ISO-2**]**,** [30 mg, **Analog8_ISO-3**] and [50 mg, **Analog8_ISO-4**].

Note: Diastereomers were separated by SFC purification; Stereochemical assigment was done arbitrarily.

<u>Analog8_ISO-1</u>

^{1}**H NMR** (400 MHz, DMSO-D$_6$) δ ppm 8.48 (d, J = 8.2 Hz, 1H), 8.13 (t, J = 6 Hz, 1H), 7.95-7.90 (m, 1H), 7.79 (d, J = 8 Hz, 1H), 7.59 (d, J = 7.2 Hz, 1H), 7.52-7.43 (m, 3H), 7.08 (d, J = 8.5 Hz, 2H), 6.81 (d, J = 8.6 Hz, 2H), 4.10 (d, J = 5.8Hz, 2H), 4.05-3.95 (m, 1H), 3.69 (s, 3H), 2.95-2.80 (m, 2H), 2.45-2.30 (m, 2H), 1.95-1.90 (m, 2H), 1.41 (d, J = 6.4 Hz, 3H).

LC-MS: *m/z* = 389 $[M + H]^+$; **HPLC**: 99.73%; **ee**>99%

<u>Analog8 ISO-2</u>

^{1}H NMR (400 MHz, DMSO-D_6) δ ppm 8.48 (d, J = 5.6 Hz, 1H), 8.13 (bs, 1H), 7.94-7.88 (m, 1H), 7.79 (d, J = 8 Hz, 1H), 7.59 (d, J = 6.8 Hz, 1H), 7.53-7.42 (m, 3H), 7.08 (d, J = 8.2 Hz, 2H), 6.81 (d, J = 8.2 Hz, 2H), 4.11 (bs, 2H), 4.05-3.95 (m, 1H), 3.69 (s, 3H), 2.95-2.78 (m, 2H), 2.67 (t, J = 8.6 Hz, 1H), 2.45-2.32 (m, 2H), 2.00-1.90 (m, 2H), 1.42 (d, J = 6.3 Hz, 3H).

LC-MS: *m/z* = 389 $[M + H]^+$; **HPLC**: 99.02%; **ee**>99%

<u>Analog8 ISO-3</u>

^{1}H NMR (400 MHz, DMSO-D_6) δ ppm 8.45 (d, J = 7.7 Hz, 1H), 8.17 (t, J = 5.8 Hz, 1H), 7.92-7.89 (m, 1H), 7.78 (d, J = 8 Hz, 1H), 7.61 (d, J = 6.9 Hz, 1H), 7.55-7.43 (m, 3H), 7.11 (d, J = 8.5 Hz, 2H), 6.84 (d, J = 8.5 Hz, 2H), 4.18-4.16 (m, 2H), 4.02 (q, J_{AB}=6.4 Hz, J_{AC}=12.8 Hz, 1H), 3.72 (s, 3H), 2.93-2.87 (m, 2H), 2.55-2.45 (m, 3H), 1.95-1.85 (m, 2H), 1.41 (d, J = 6.4 Hz, 3H).

LC-MS: *m/z* = 389 $[M + H]^+$; **HPLC**: 99.76%; **ee**>97%

<u>Analog8 -ISO-4</u>

^{1}H NMR (400 MHz, DMSO-D_6) δ ppm 8.46-8.42 (m, 1H), 8.18 (t, J = 5.8 Hz, 1H), 7.95-7.90 (m, 1H), 7.78 (d, J = 7.9 Hz, 1H), 7.61 (d, J = 6.6 Hz, 1H), 7.52-7.43 (m, 3H), 7.11 (d, J = 8.4 Hz, 2H), 6.85 (d, J = 8.6 Hz, 2H), 4.16 (d, J = 4.3 Hz, 2H), 4.05-3.95 (m, 1H), 3.72 (s, 3H), 2.95-2.85 (m, 2H), 2.55-2.45 (m, 3H), 1.99-1.80 (m, 2H), 1.41 (d, J = 6.4 Hz, 3H).

LC-MS: *m/z* = 389 [M + H]$^+$; **HPLC**: 99.72%; **ee**>99%

Syntheses of (*R*)-2-(4-methoxyphenyl)-1-(4-(1-(naphthalen-1-yl)ethyl)piperazin-1-yl)ethan-1-one [Analogue9_ISO-1] and (*S*)-2-(4-methoxyphenyl)-1-(4-(1-(naphtha-len-1-yl)ethyl)piperazin-1-yl)ethan-1-one [Analog 9_ISO-2]:

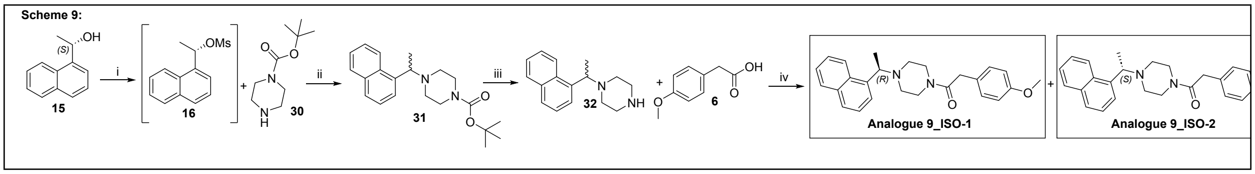

Reagent and conditions: (i-ii) Ms_2O, DIPEA, DCM, 0°C-rt, 20h; (iii) HCl (2.0 M in diethyl ether), 0°C-rt, 16h; (iv) 1) HATU, DIPEA, DMF, 0°C-rt, 16h; 2) SFC separation

Synthesis of *tert*-butyl-4-(1-(naphthalen-1-yl)ethyl)piperazine-1-carboxylate (**31**):

Synthesis of *tert*-butyl-4-(1-(naphthalen-1-yl)ethyl)piperazine-1-carboxylate (**31**) has been achieved by following the similar procedure described for compound 4 (Scheme 1) by using (*S*)-1-(naphthalen-1-yl)ethan-1-ol (200 mg, 1.16 mmol) and *tert*-Butyl piperazine-1-carboxylate (432 mg, 2.32 mmol) to afford tert-butyl-4-(1-(naphthalen-1-yl)ethyl)piperazine-1-carboxylate (**31**) (250 mg, 63%) as light-yellow gum. The crude product was carried forwarded to the next step without further purification.

Note: Although synthesis started with pure chiral synthon (**15**), during substitution reaction racemization observed.

^{1}H NMR (400 MHz, DMSO-D$_6$) δ ppm 8.42 (d, J = 8.1 Hz, 1H), 7.92-7.90 (m, 1H), 7.80 (d, J = 8Hz, 1H), 7.57-7.40 (m, 4H), 4.20-4.15 (m, 1H), 3.26 (bs, 4H), 2.42 (bs, 2H), 2.31 (bs, 2H), 1.40-1.30 (m, 12H)

LC-MS: *m/z* = 341.44 $[M + H]^+$

<u>1-(1-(Naphthalen-1-yl)ethyl)piperazine (**32**):</u>

To a stirred ice-cooled solution of *tert*-butyl-4-(1-(naphthalen-1-yl)ethyl)piperazine-1-carboxylate (**31**) (250 mg) in diethyl ether (2 mL) was added a solution of HCl (2.0 M in diethyl ether). Resultant mixture was slowly warmed to room temperature and stirred for 16 hours. After completion, volatiles were removed under reduced pressure and residue was triturated with *n*-pentane to afford 1-(1-(naphthalen-1-yl)ethyl)piperazine HCl salt (**32**)(150 mg, 85%) as light-yellow solid. The crude product was carried to the next step without further purifiication.

LC-MS: *m/z* = 241.45 $[M + H]^+$

<u>Synthesis of (*R*)-2-(4-methoxyphenyl)-1-(4-(1-(naphthalen-1-yl)ethyl)piperazin-1-yl)ethan-1-one [**Analog9_ISO-1**] & (*S*)-2-(4-methoxyphenyl)-1-(4-(1-(naphthalen-1-yl)ethyl)piperazin-1-yl)ethan-1-one [**Analog9_ISO-2**]:</u>

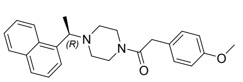

Analogue 9_ISO-1

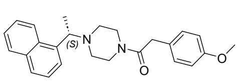

Analogue 9_ISO-2

To a stirred ice-cooled solution of 2-(4-methoxyphenyl)acetic acid (123 mg, 0.74 mmol) in DMF (3 mL) under argon atmosphere was added HATU (422 mg, 1.11 mmol) in one-portion followed by dropwise addition of DIPEA (0.54 mL, 3.08 mmol). Resultant mixture was stirred at room temperature for 20 min. compound 32 (148 mg, 0.617 mmol) was added to the reaction mixture in portions and stirred at room temperature for 16 hours. After completion of the reaction, the mixture was diluted with ice-cooled water and extracted with ethyl acetate. Combined organic layer was washed with brine, dried over anhydrous sodium sulphate, filtered and concentrated under reduced pressure. Crude product thus obtained was purified by ISCO column to afford racemic 2-(4-methoxyphenyl)-1-(4-(1-(naphthalen-1-yl)ethyl)piperazin-1-yl)ethan-1-one (**Analog 9**) (110 mg, 46%) as off-white solid. This material was further subjected to chiral SFC separation to afford (*R*)-2-(4-methoxyphenyl)-1-(4-(1-(naphthalen-1-yl)ethyl)piperazin-1-yl)ethan-1-one (**Analog 9_ISO-1,** 41 mg) as off white solid and (*S*)-2-(4-methoxyphenyl)-1-(4-(1-(naphthalen-1-yl)ethyl)piperazin-1-yl)ethan-1-one (**Analogue 9-ISO-2**, 50 mg) as off white solid.

Note: Enantiomers were separated by SFC purification; Stereochemistry was arbitrarily assigned.

Analog9-ISO-1

^{1}H NMR (400 MHz, DMSO-D_6) δ ppm 8.45-8.41 (m, 1H), 7.93-7.89 (m, 1H), 7.80 (d, J = 8 Hz, 1H), 7.58-7.42 (m, 4H), 7.10 (d, J = 8.5 Hz, 2H), 6.83 (d, J = 8.6 Hz, 2H), 4.20-4.10 (m, 1H), 3.71 (s, 3H), 3.58 (s, 2H), 3.60-3.30 (m, 5H), 2.45-2.25 (m, 3H), 1.38 (d, J = 6.6 Hz, 3H)

LC-MS: *m/z* = 389 $[M + H]^+$; **HPLC**: 99.78%; **ee**>99%

Analog9-ISO-2

^{1}H NMR (400 MHz, DMSO-D_6) δ 8.41 (d, J = 8.1 Hz, 1H), 7.94-7.90 (m, 1H), 7.80 (d, J = 8 Hz, 1H), 7.58-7.44 (m, 4H), 7.10 (d, J = 8.4 Hz, 2H), 6.83 (d, J = 8.4 Hz, 2H), 4.20-4.10 (m, 1H), 3.71 (s, 3H), 3.58 (s, 2H), 3.60-3.30 (m, 4H), 2.45-2.25 (m, 4H), 1.38 (d, J = 6.5 Hz, 3H)

LC-MS: *m/z* = 389 $[M + H]^+$; **HPLC**: 99.20%; **ee**>99.

Syntheses of (*R*)-2-(4-methoxyphenyl)-N-(1-(1-(naphthalen-1-yl)ethyl)piperidin-4-yl)acetamide [**Analo-g10_ISO-1**] and (S)-2-(4-methoxyphenyl)-N-(1-(1-(naph-thalen-1-yl)ethyl)piperidin-4-yl)acetamide [**Analog10_ISO-2**]

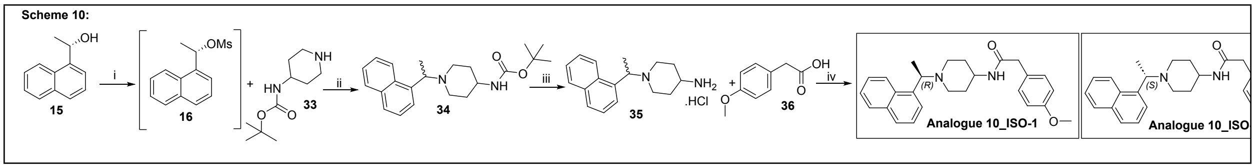

Reagent and conditions: (i-ii) Ms_2O, DIPEA, MS 4A°, DCM, 0°C-rt, 20h; (iii) HCl (2.0 M in diethyl ether), 0 -25°C, 16h; (iv) 1) HATU, DIPEA, DMF, 0°C-rt, 16h; 2) SFC separation.

tert-Butyl-(1-(1-(naphthalen-1-yl)ethyl)piperidin-4-yl)carbamate (**34**):

Synthesis of *tert*-butyl-(1-(1-(naphthalen-1-yl)ethyl)piperidin-4-yl)carbamate (**34**) was achieved by following the similar procedure described for compound 4 (Scheme 1) using (*S*)-1-(naphthalen-1-yl)ethan-1-ol (**15**) (300 mg, 1.742 mmol) and tert-butyl piperidin-4-ylcarbamate (**33**) (697 mg, 3.48 mmol) to afford tert-butyl-(1-(1-(naphthalen-1-yl)ethyl)piperidin-4-yl)carbamate (**34**) (200 mg, 32%) as pale yellow liquid.

¹H NMR (400 MHz, DMSO-D$_6$) δ ppm 8.41 (d, J = 6.4 Hz, 1H), 7.90 (d, J = 7.3 Hz, 1H), 7.78 (d, J = 7.6 Hz, 1H), 7.53-7.43 (m, 4H), 6.70 (d, J = 5.4 Hz, 1H), 4.17-4.12 (m, 1H), 3.30-3.20 (m, 2H), 3.00-2.80 (m, 1H),2.73-2.67 (m, 1H), 2.10-1.90 (m, 2H), 1.80-1.65 (m 1H), 1.65-1.55 (m, 1H), 1.40-1.25 (m, 12H).

LC-MS: *m/z* = 355 [M + H]$^+$

<u>1-(1-(Naphthalen-1-yl)ethyl)piperidin-4-amine hyrdochloride (**35**):</u>

Synthesis of 1-(1-(naphthalen-1-yl)ethyl)piperidin-4-amine hydrochloride (**35**) was achieved by following the similar procedure described for (**Int-32**, **Scheme 9**) using tert-butyl-(1-(1-(naphthalen-1-yl)ethyl)piperidin-4-yl)carbamate (**34**) (210 mg, 0.592 mmol) to afford 1-(1-(naphthalen-1-yl)ethyl)piperidin-4-amine hydrochloride (**35**) (120 mg, 79%) as pale yellow solid.

LC-MS: *m/z* = 255.41 [M + H]$^+$

<u>Synthesis of (*R*)-2-(4-methoxyphenyl)-N-(1-(1-(naphthalen-1-yl)ethyl)piperidin-4-yl)acetamide (**Analog 10_ISO-1**) and (S)-2-(4-methoxyphenyl)-N-(1-(1-(naphthalen-1-yl)ethyl)piperidin-4-yl)acetamide (**Analog 10-ISO-2**):</u>

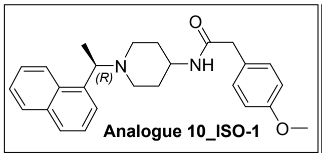

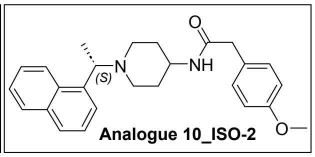

It has been synthesized following similar procedure described for Analog 9 using compound **35** (150 mg, 0.59 mmol) and 2-(4-methoxyphenyl)acetic acid (**36**) (118 mg, 0.708 mmol) to afford

racemic 2-(4-methoxyphenyl)-N-(1-(1-(naphthalen-1-yl)ethyl)piperidin-4-yl)acetamide (**Analog 10**) (120 mg, 50%) which was further subjected to chiral separation by chiral SFC to afford (*R*)-2-(4-methoxyphenyl)-N-(1-(1-(naphthalen-1-yl)ethyl)piperidin-4-yl)acetamide (**Analog 10_ISO-1,** 41 mg) and (S)-2-(4-methoxyphenyl)-N-(1-(1-(naphthalen-1-yl)ethyl)piperidin-4-yl)acetamide (**Analog 10-ISO-2,** 56 mg) as white solids.

Note: Enantiomers were separated by SFC purification; Stereochemistry was arbitrarily assigned.

^{1}H NMR (400 MHz, DMSO-D_6) δ 8.45 (d, J = 8.8 Hz, 1H), 7.91-7.85 (m, 2H), 7.79 (d, J = 8Hz, 1H), 7.54-7.43 (m, 4H), 7.13 (d, J = 8.6 Hz, 2H), 6.82 (d, J = 8.6 Hz, 2H), 4.20-4.10 (m, 1H), 3.70 (s, 3H), 3.50-3.40 (m, 1H), 3.26 (s, 2H), 2.96-2.92 (m, 1H), 2.72-2.70 (m, 1H), 2.15-2.05 (m, 2H) 1.75-1.55 (m, 2H), 1.39 (d, J = 6.6Hz, 3H), 1.35-1.29 (m, 2H)

LC-MS: *m/z* = 403 $[M + H]^+$; **HPLC**: 99.83%; **ee**>99%

^{1}H NMR (400 MHz, DMSO-D_6) δ 8.45 (d, J = 8.9 Hz, 1H), 7.91-7.85 (m, 2H), 7.79 (d, J = 8Hz, 1H), 7.53-7.43 (m, 4H), 7.12 (d, J = 8.5 Hz, 2H), 6.82 (d, J = 8.5 Hz, 2H), 4.20-4.05 (m, 1H), 3.70 (s, 3H), 3.55-3.45 (m, 1H), 3.26 (s, 2H), 2.96-2.92 (m, 1H), 2.72-2.70 (m, 1H), 2.20-2.00 (m, 2H) 1.75-1.55 (m, 2H), 1.40 (d, J = 6.6Hz, 3H), 1.38-1.20 (m, 2H)

LC-MS: *m/z* = 403 $[M + H]^+$; **HPLC**: 99.81%; **ee**>99%
